# Grapes Ready for Picking: Investigating the Readiness of Precision Viticulture Datasets for Scene Reconstruction

**DOI:** 10.3390/s26216708

**Published:** 2026-10-23

**Authors:** Michał Jan Włodarczyk, Katarzyna Wasielewska-Michniewska, Przemysław Hołda, Maria Ganzha, Marcin Paprzycki, Nico Heider, Martin Schieck, Bogdan Franczyk, Tasuku Makabe, Iori Yanokura, Kei Okada

**Affiliations:** 1 Systems Research Institute, Polish Academy of Sciences, 01-447 Warsaw, Poland; katarzyna.wasielewska@ibspan.waw.pl (K.W.-M.); pholda@ibspan.waw.pl (P.H.); maria.ganzha@ibspan.waw.pl (M.G.); paprzyck@ibspan.waw.pl (M.P.); 2 Smart Farming Lab, Leipzig University, Grimmaische Straße 12, 04109 Leipzig, Germany; nico.heider@infai.org (N.H.); schieck@wifa.uni-leipzig.de (M.S.); franczyk@wifa.uni-leipzig.de (B.F.); 3 JSK Robotics Laboratory, University of Tokyo, 7-3-1 Hongo, Bunkyo-ku, Tokyo 113-8656, Japan; makabe@jsk.imi.i.u-tokyo.ac.jp (T.M.); yanokura@jsk.imi.i.u-tokyo.ac.jp (I.Y.); k-okada@jsk.t.u-tokyo.ac.jp (K.O.)

**Keywords:** precision viticulture, scene reconstruction, 3D Gaussian splatting, structure from motion, dataset characterization, UAV imagery, agricultural robotics

## Abstract

Public datasets of vineyard imagery were collected for detection, segmentation and disease mapping and are now proposed as a source for 3D scene reconstruction. Their documentation rarely states the properties that reconstruction depends on, such as view overlap, parallax and frame spacing, so it is unclear which can be reconstructed. This study measures that for the publicly available vineyard datasets. A structured search identifies eighteen datasets. Ten are characterized under a schema of thirty-six documented and measured properties, and only six contain overlapping views. Every sequence that registers is reconstructed with one fixed pipeline of structure from motion and 3D Gaussian splatting, giving twenty-seven reconstructions on two held-out splits. Registration is decided by the capture rather than by the dataset; novel-view quality varies as much within a dataset as between datasets; and frame spacing is the one capture property that limits reconstruction. Three preprocessing steps are tested as ablations on the failure cases. Band alignment restores registration, whereas dehazing a backlit pass and masking the chassis of a ground robot give no improvement that this study can resolve. The schema and pipeline apply to any vineyard dataset, and the results are published as an open online catalog.

## 1. Introduction

The rapid advancement of smart agriculture has established precision viticulture (PV) as a critical domain for integrating Artificial Intelligence (AI) and Unmanned Aerial Vehicle (UAV) systems into daily vineyard operations [1]. The core objective of PV is to optimize grape production, reduce resource consumption, and enhance sustainability through continuous monitoring and management [2,3,4,5,6,7,8]. To achieve this, modern PV relies heavily on automated systems powered by machine learning, sensors and drone imagery [9]. However, the efficacy of these advanced models is fundamentally dictated by the availability and quality of the data used to train them. Consequently, robust datasets have become the foundational backbone of PV.

Although numerous PV-related datasets have been introduced to the research community in recent years, the majority were designed following the needs of a specific deployment or with specific, often narrow, primary objectives in mind, such as 2D object detection (e.g., [10,11,12]), basic disease classification (e.g., [13,14]) or simple semantic segmentation (e.g., [10,11,15]).

The history of the available datasets explains this narrow focus. The first public datasets, such as the Embrapa Wine Grape Instance Segmentation Dataset (WGISD) [15] and GrapeCS-ML [11], consist mainly of handheld photographs taken in 2017 and 2018 for cluster detection, instance segmentation, and phenological staging. From 2021 onward, a single estate in Tomiño, Spain, added UAV video, multispectral imagery and light detection and ranging (LiDAR) scans of the same vineyard block through datasets such as Bodegas Terras Gauda (BTG) [16], Multispectral Botrytis (Botrytis) [17], GrapeMOTS [18], VineLiDAR [19], and EscaYard [13]. Each of them serves a specific task (e.g., cluster counting, disease mapping or canopy geometry). Ground robots followed in 2022 and 2023, with the Bacchus Long-Term (BLT) dataset [20] recording a full season in Greece and three weeks in the UK. As a result, most of these datasets answer a 2D question, and the properties that a 3D method needs, such as view overlap, parallax and frame spacing, were rarely recorded.

In the same period, scene reconstruction evolved from a specialist photogrammetric task into one that consumes ordinary video. In 2020, Neural Radiance Fields (NeRF) [21] showed that a photorealistic scene model can be optimized from posed images alone. Three years later, 3D Gaussian splatting (3DGS) [22] made such reconstructions fast enough to render in real time. Because both methods recover their camera poses through structure from motion (SfM), their only input is a set of overlapping images of the scene. Consequently, reusing existing vineyard datasets for reconstruction is a natural step, and recent work already builds Gaussian-splatting assets of crops and greenhouses, although from newly collected imagery [23,24,25]. However, no study has yet tested which of the existing vineyard datasets can actually be reconstructed.

**Figure 1:**
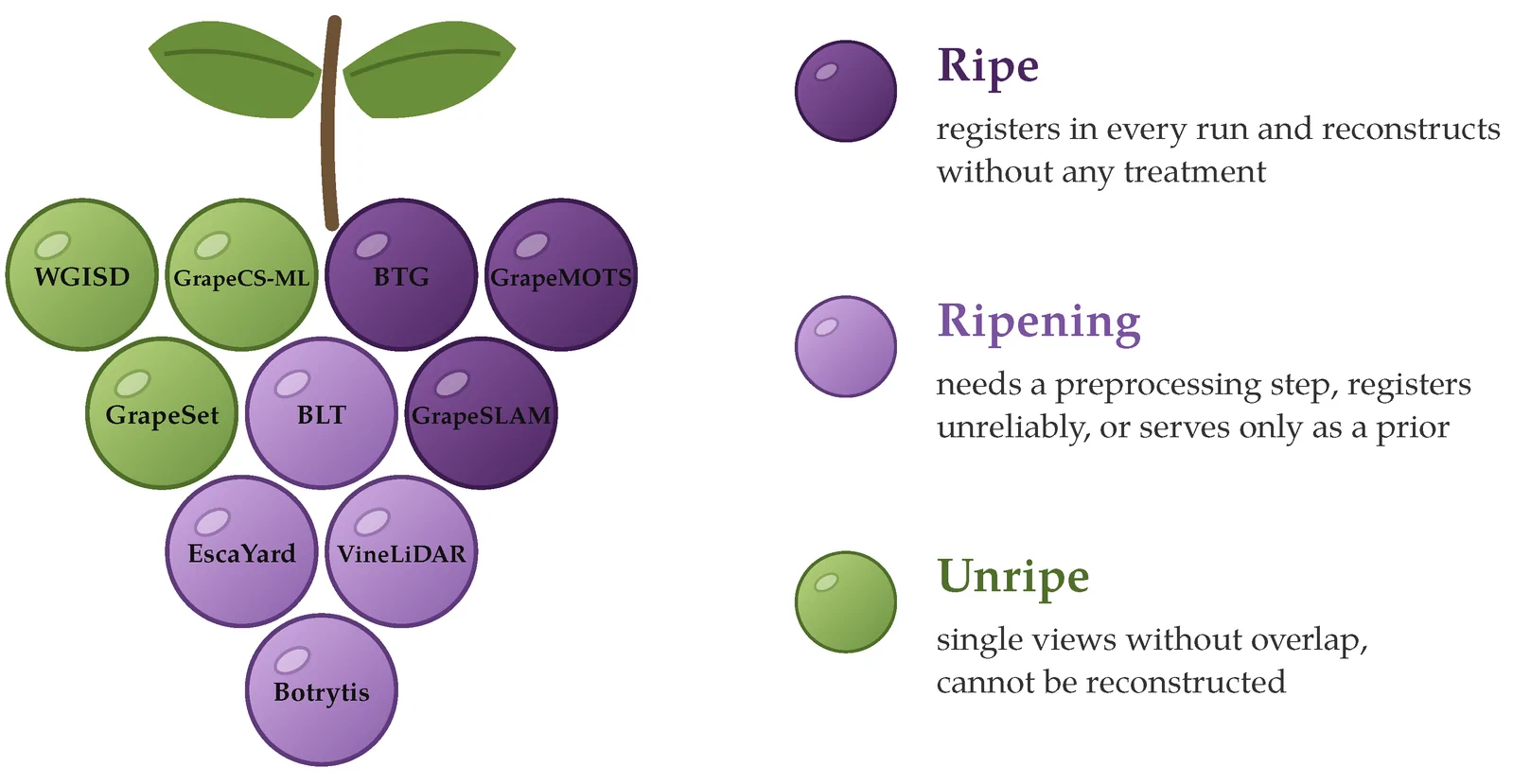
Readiness of the ten characterized vineyard datasets for scene reconstruction, drawn as a bunch, i.e., the end result of the characterization and reconstruction procedure proposed in this paper. Section 6.1 explains how a dataset is assigned to a class and gives the measured values behind each class.

Reuse also carries a risk that is easy to overlook. A dataset recorded for detection or segmentation may lack exactly the properties that reconstruction depends on, and its documentation rarely states whether it does. Avoiding a new recording campaign for every reconstruction need is a real saving only once this risk has been measured. To provide that measurement, we make the following four contributions:

- A characterization schema of 36 documented and measured properties that describes a dataset by what reconstruction needs rather than by its original task and that can be applied to any existing or new dataset.
- A snapshot of the publicly available vineyard datasets as of October 2026, showing that only 6 of the 18 datasets found by a structured search contain overlapping views (Figure 1) and that none of them documents its parallax or viewpoint coverage. Published online, https://dragons-project.github.io/grapes-ready-for-picking/ (accessed on 31 August 2026) the snapshot guides dataset users with a decision procedure for selecting reconstructable data, and it gives creators and users recommendations for recording and reuse that rest on measured results.
- A reconstruction study in which we reconstruct every sequence that registers with the same 3DGS pipeline, yielding 27 reconstructions. The study shows that registration depends on how a dataset was captured rather than on which dataset it is, and that frame spacing, not image resolution, limits reconstruction.
- An ablation of three targeted preprocessing steps for the datasets that fail to register or reconstruct with visible artifacts, showing that multispectral band alignment restores registration, whereas dehazing of a backlit sequence and masking of a robot chassis give no improvement that this study can resolve.

The remainder of this paper is organized as follows. Section 2 reviews dataset surveys, scene reconstruction in agriculture, and reconstruction benchmarks. Section 3 describes the dataset search, the characterization schema, and the characterized datasets. Section 4 reconstructs every registered sequence under one fixed pipeline and derives the capture requirements, and Section 5 evaluates the preprocessing steps on the failure cases. Section 6 summarizes the readiness of each dataset, the gaps in the available data, and the resulting recommendations, Section 7 discusses the limitations of the study, and Section 8 concludes the paper.

## 2. Related Work

### 2.1. Dataset Surveys and Documentation

The vineyard datasets themselves are the subject of Section 3, while this section reviews the prior work closest to this study. Surveys of public agricultural datasets catalogue what exists, ranging from field datasets for precision agriculture [26] and field image collections [27] to datasets for robot localization and mapping in agriculture and forestry [28], as well as reviews of grapevine datasets [29] and close-range aerial imagery in viticulture [7]. However, these surveys describe each dataset by its annotations, sensors, and original task rather than by the geometric properties that reconstruction depends on. Recent multi-view crop datasets, such as GroMo25 [30] and TomatoMAP [31], record plants from several angles on purpose, but they target controlled phenotyping setups rather than field-scale scenes. In parallel, documentation standards such as Datasheets for Datasets [32] and the Findable, Accessible, Interoperable and Reusable (FAIR) principles [33] ask dataset creators to state how their data were collected and how they can be reused. Our characterization schema checks exactly these statements against the data.

### 2.2. Scene Reconstruction in Agricultural Environments

In viticulture, 3D modeling has long relied on classical SfM and photogrammetry, which are mature tools for metric extraction, such as the Leaf Area Index [34] or canopy volume, rather than for reusable, navigable 3D assets. NeRF [21] and 3DGS [22] changed this by producing photorealistic scene models from posed images. Plant3R [35], Cotton3DGaussians [24] and PlantGaussian [25] model foliage and fruit in detail, but only for single plants or controlled, small-scale setups. Orchard-scale systems, such as InspectGaussian [36] for inspection robots and AgriGS-SLAM [37] for visual–LiDAR mapping across seasons, extend 3DGS to larger scenes, but they rely on imagery recorded for that purpose. The closest precedent to our work is GreenhouseSplat [23], which builds Gaussian-splatting assets of cucumber rows from newly collected red, green and blue (RGB) images and integrates them into simulation based on the Robot Operating System (ROS), although only in a greenhouse. None of these studies asks whether existing field datasets, recorded for other purposes, can serve as input, which is the question this paper addresses for vineyards.

### 2.3. Reconstruction Benchmarks and Evaluation

Reconstruction benchmarks take the opposite view to dataset surveys, since they evaluate methods on scenes captured specifically for that purpose, with overlap and coverage controlled by their authors. General benchmarks such as Tanks and Temples [38], ETH3D [39], and Mip-NeRF 360 [40] established this practice, and UAV3DCrop [41] recently extended it to repeated multi-angle UAV surveys of field crops. Novel-view synthesis benchmarks commonly hold out every eighth image of the training trajectory, and UAV3DCrop holds out every tenth. Both settings test interpolation between neighboring views rather than rendering away from them, a limitation that UAV3DCrop itself acknowledges. To measure the latter, Nerfbusters [42] evaluates on a second separately recorded camera path, and the same concern underlies the block split of Section 4.1. This study combines the perspectives of surveys and benchmarks by applying a benchmark-style evaluation with one fixed pipeline to agricultural data that was never captured for reconstruction.

## 3. Publicly Available Datasets

In this section, we survey the publicly available vineyard datasets, that is, the datasets of vineyard imagery identified by our search that can be downloaded or requested today, and we refer to them collectively as the record. To do so, we first find the available datasets through a structured search, then keep those that at least partially meet the requirements of reconstruction or are widely reused, and finally measure them under one schema of documented and measured properties. The result is an inventory of what exists and what it contains, not a review of the literature that uses it.

### 3.1. Dataset Selection Process

During the development of PV solutions, a number of datasets have been published for potential reuse. To identify them, we performed a structured manual search and screening process. Although the selection process was not conducted as a formal systematic literature review (SLR), it followed a structured and reproducible screening logic inspired by systematic review practices. We examined the relevant scientific literature and publicly available repositories to identify datasets applicable to viticulture-related research. We then assessed the datasets found according to relevance, accessibility, availability of documentation and suitability. The goal was to select candidate datasets that could be analyzed further and used in the experiments of this work. The basic inclusion criterion was that a dataset contains PV-related images or videos, which excludes datasets that provide only physiological or meteorological measurements in tabular form. Therefore, the dataset identification was based on a combination of literature-informed identification, repository-based searches, and manual assessment of dataset suitability (see Table 1). The search combined a large language model (LLM) research agent with manual citation network exploration in three stages. First, we obtained an initial seed of papers and datasets from the Research mode of Claude (Anthropic), running the Claude Opus 5 model, which searches the web and returns a report with its sources. Second, we expanded this seed along two independent pipelines. In the human pipeline, we added the seed papers to a collection in ResearchRabbit [43], a literature-mapping tool that recommends publications connected to a collection through its citation network. The tool offers three kinds of recommendations, namely earlier work (the references of the collected papers), later work (the papers that cite them), and similar work (papers linked by co-citation, bibliographic coupling, or content similarity). In each pass, we screened these recommendations by title and abstract, added the relevant papers to the collection, and started the next pass from the enlarged collection, so that the search snowballed backward and forward through the literature. We repeated these passes until one added no new papers, which occurred after five passes. In the LLM pipeline, we continued with a multi-turn research conversation with Claude Opus 5, in which each pass refined the queries of the previous one, and we applied the same stopping rule, which ended this pipeline after three passes. Finally, we merged the candidates from both pipelines and removed duplicates, and a human author verified every candidate proposed by the LLM before it entered Table 1. The GrapeSLAM dataset [44], for instance, was surfaced by the LLM pipeline from the record of a dataset already in the collection, and was then verified and characterized in the same way as the rest. The search was closed in October 2026. We limited the final selection to datasets that at least partially fulfill the requirements for scene reconstruction (e.g., multi-view images with ground-truth information), together with datasets that are often reused and referenced in the literature. Of the 18 datasets listed in Table 1, we characterize 10 in full in the remainder of this section. The other eight, GrapeNet [10], GRAPE Leaf Diseases (GLD) [45], Grape Leaf Dataset for Leaf Health Classification and Disease Detection (GLHD) [14], Leaf Diseases Dataset (LDD) [46], Grape-Instance-Segmentation-For-Viticulture (GISV) [47], UAV-based Vine Leaf Instance Segmentation (UAV-VLIS) [48], Synthetic RGB Data for Grapevine Detection (SynthRGB) [49] and the Grape bunch and vine trunk dataset (GBVT) [50], consist of single or synthetic images annotated for classification, detection, or segmentation, which lack the overlapping views that requirement (ii) demands. The caption of Table 1 gives the reason for each exclusion. Four of the ten characterized datasets, WGISD, GrapeCS-ML, GrapeSet and EscaYard, also consist mainly of photographs, but their documentation describes more than one view of the same scene, namely a video in the WGISD repository, repeated rounds of the same Merlot vines in GrapeCS-ML, four views of each bunch in GrapeSet, and UAV point clouds of the photographed plants in EscaYard. Whether they meet requirement (ii) could therefore be decided only by measurement, and they were characterized to make that measurement. Although the list is not exhaustive, Section 6.4 describes an online catalog through which further datasets can be added as they appear.

### 3.2. Requirements for the Dataset

Ideally, for 3D scene reconstruction of vineyards, a good dataset needs to support not just plant recognition, but the recovery of 3D geometry, spatial structure, and camera/viewpoint information. An eligible dataset (i) contains imagery acquired in real vineyard environments; (ii) provides multiple views of the same vineyard scene or plant structures; (iii) contains sufficient spatial, visual and temporal information; (iv) provides adequate documentation of the acquisition setup and sensor characteristics; (v) is publicly accessible or obtainable from a documented source; and (vi) shows plants with visible structural features in rows rather than as isolated objects. Diversity of cultivars, canopy densities and growth stages is a property of the record as a whole rather than of one dataset, and it is assessed in the characterization rather than required of each dataset. Requirements (i), (v) and (vi) can be verified directly from documentation, whereas requirements (ii–iv) can only be established by measurement. Not every identified dataset satisfies all of these requirements, but some of them become usable after the preprocessing steps of Section 5.

### 3.3. Characterization Schema

Each characterized dataset is described by the 36 properties listed in Table 2, which are grouped by four questions: what scene was captured, how it was observed, what was recorded, and what the recording yields when it is used for reconstruction. The first three groups record decisions made by the dataset authors and can be filled from documentation. The fourth group cannot, because no dataset reports how much of its imagery registers, how much parallax its frames contain, or how much of each frame remains static. Since these are precisely the properties that decide whether reconstruction is possible at all, we measure them here.

To obtain the measured properties, we apply one procedure to all datasets. We reduce every sequence to at most 200 frames at no more than 2 frames per second and resize the frames to a width of at most 1600 pixels. The resulting frame lists are reused unchanged in all later experiments. Sharpness, exposure and static content are computed directly from the frames, while the geometric properties are read with a single SfM configuration that is identical for every sequence. A sequence that does not register is treated as a result rather than as a missing value. Sequences whose outcome changes between runs are repeated five times, and their registration stability is reported as a success rate. SfM serves here only as an instrument, since reconstruction itself is assessed separately in the following section. Because the dataset is the unit of reporting while the sequence is the unit of measurement, we report each measured value as the median over a dataset’s sequences together with its range. All parameters and software versions are listed in Appendix A and in the code repository.

### 3.4. Characterized Datasets

Table A3 and Table A4 list the documented and measured properties of the ten characterized datasets in Appendix C, and the descriptions below focus on what each dataset offers for reconstruction and where its capture choices limit it.

The BTG dataset [16] provides the dense frame overlap that SfM requires: a low-altitude UAV flight at 3 m along four rows of a Loureiro block in Tomiño, recorded at 60 fps with the camera tilted 60° toward the canopy. Because the flight precedes harvest by two months, the clusters remain largely hidden by foliage, and because each pass covers only one side of a row, the resulting model is a façade rather than a volume. Although the data paper describes the parts of each clip as contiguous, they are separate recordings that register as separate models, which makes the part the unit of reconstruction here. No camera trajectory accompanies the videos.

The Botrytis dataset [17] captures the same block on harvest day with four flights of a five-band MicaSense RedEdge 3 at nadir, 30° and 45°, at one-second intervals and roughly 80% overlap. Geometrically, it is the richest capture in the record, with the widest viewing angles and the only surveyed ground control points (GCPs). However, its five bands are recorded through separate lenses, so a usable color image requires band alignment before any reconstruction, a problem addressed among the failure cases below. Finally, the data paper states a flight height of 30 m, whereas the EXIF altitudes imply 16 to 20 m.

GrapeMOTS [18] returns to the block at harvest with a Phantom 4 RTK at 3 m, when the clusters are mature, and covers both sides of the canopy. Its eleven videos form two groups with different geometry (three frontal passes along rows of about 110 m, and eight multi-angle orbits of single vines, which the data paper describes as path-planned views). While the orbits offer the widest viewpoint coverage of any UAV sequence, they reconstruct a single plant each, so the row passes constitute the field-scale material. One pass was flown against a low sun and suffers from veiling glare and a lens ghost, which Section 5.2 examines as a failure case. As with BTG, no camera trajectory is deposited with the videos.

GrapeSLAM [44] adds 24 videos from the same estate, recorded at 1 to 3 m under varying illumination for visual localization, each accompanied by the drone’s flight log. Although the data paper describes the log as RTK, its own columns report standard Global Positioning System (GPS) positioning. It nevertheless aligns to the reconstruction with a trajectory error of 5.6 cm, which makes GrapeSLAM the only Tomiño capture with a usable camera trajectory.

The BLT dataset [20] is the only ground robot dataset among those characterized and the only one with seasonal temporal structure. A Thorvald II traversed the same corridors of a vineyard at Ktima Gerovassiliou, Greece, in ten sessions between March and September 2022, recording with two RGB-D cameras, one looking forward and one sideways, together with a 3D LiDAR, an IMU, and RTK-GNSS. Five further sessions were recorded at Riseholme, UK, two of them without cameras [51]. This seasonal coverage is unique among the characterized datasets, but it comes with a demanding capture geometry. The forward-looking camera sees the row along the direction of motion, and the robot body occupies about 29% of the UK front camera’s frame. Unlike the other characterized datasets, the data are available only after registration.

EscaYard [13] documents blocks B7 and B9 of the estate in July 2022 at plant level, with 253 geotagged smartphone photographs and per-plant esca status and cluster counts. Its UAV multispectral survey is distributed as orthomosaics and single-band point clouds rather than as frames, so the imagery available for reconstruction consists of unrelated photographs, half of which lie near a surveyed trunk.

VineLiDAR [19] supplies ten colored LiDAR point clouds of the same two blocks from three dates between September 2021 and September 2022, flown at 20 to 50 m. No imagery is shipped, so the clouds serve as geometric priors rather than as reconstruction input. Although eight of the files are georeferenced, the two from 16 September 2021, the day of the Botrytis flights, hold local coordinates, which prevents the two datasets from being registered to each other.

WGISD [15] consists of 300 handheld photographs of 5 cultivars at a winery in São Paulo, Brazil, each taken 1 to 2 m from the vines and annotated with bunch boxes and masks. As single views, these photographs cannot initialize SfM. The project repository, though not the deposit, also contains 500 consecutive frames of a demonstration video, which register in full. The deposit is licensed CC BY-NC-SA, while its README states CC BY-NC.

GrapeCS-ML [11] contributes 2327 files of 15 cultivars from three Australian vineyards, taken between January and May 2017, with berry chemistry for part of them. Four of its five sets were captured with a 640 × 480 compact camera and the fifth with phones. Although the requirement sweeps of Section 4.5 show that this resolution alone would not have prevented reconstruction, the absence of overlapping views excludes the dataset. In addition, no license is stated.

GrapeSet [12] photographs ten detached bunches on an artificial arch indoors, each combination from four sides, with masks, berry counts, and weights. The four views are obtained by rotating the bunches in front of a fixed camera rather than by moving the camera, so they are not four camera positions and do not register.

The four datasets of isolated photographs were characterized because their documentation describes more than one view of a scene (Section 3.1), and under the fixed configuration, at most 5% of their images register in two-image models, which is the measured form of requirement (ii).

### 3.5. What the Datasets Contain

**Figure 2:**
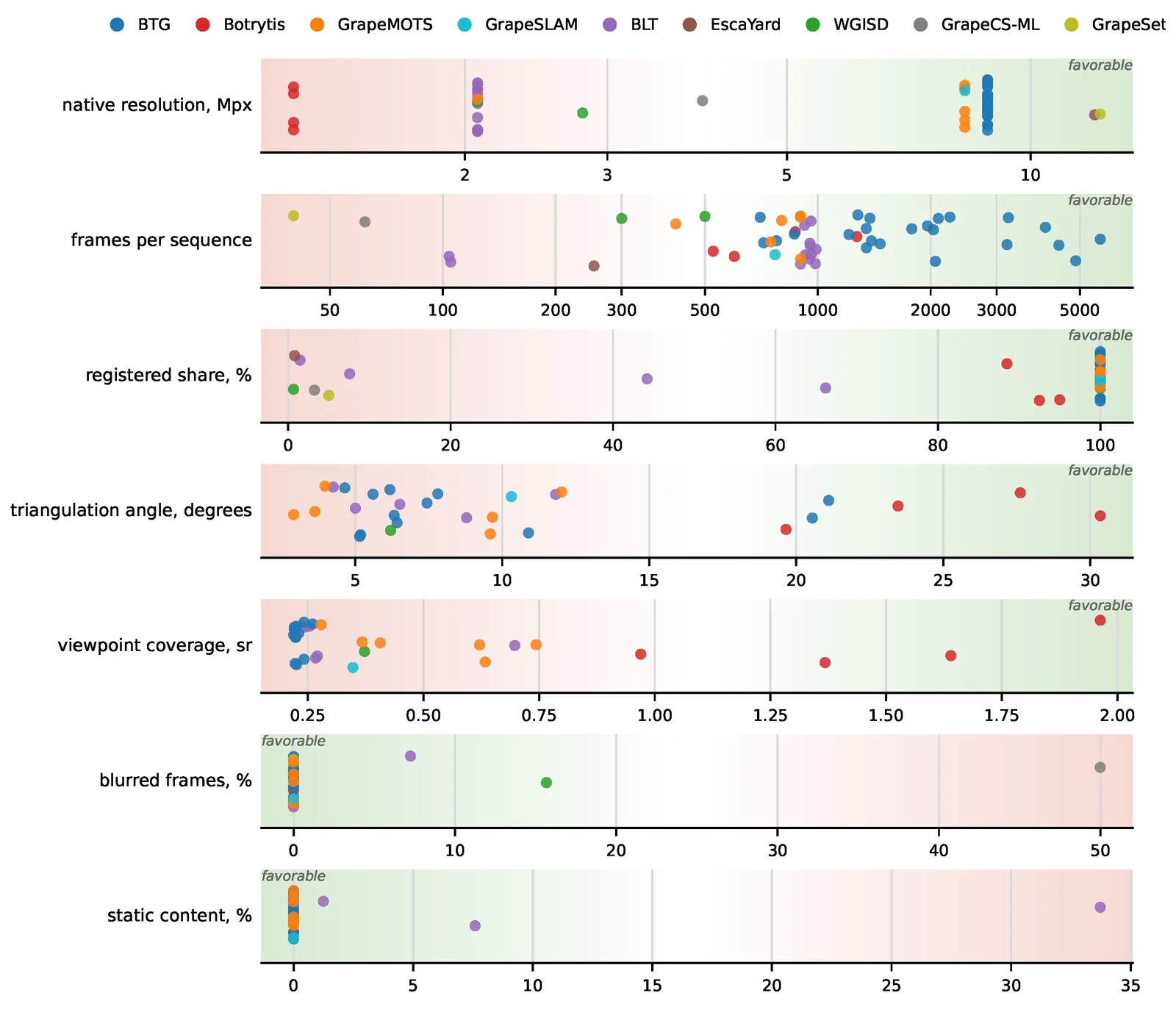
Measured quantities of the characterized sequences, one row per property and one point per sequence. The rows are the properties that reconstruction depends on native resolution, frame count, registered share, triangulation angle, viewpoint coverage, blurred frames and static content. Each row’s background then runs from red at the end that is unfavorable for reconstruction to green at the favorable end, with higher being better for the first five properties and lower for the last two.

**Figure 3:**
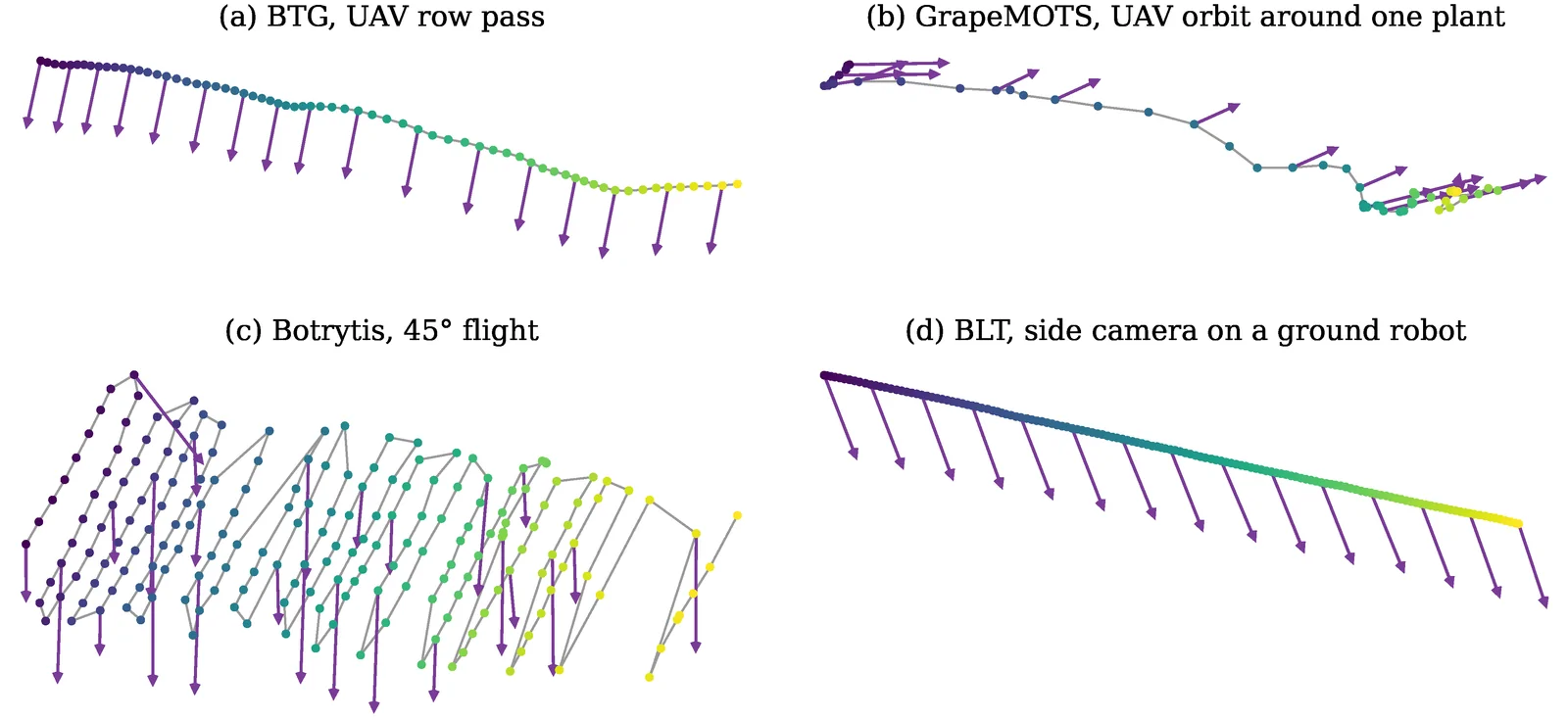
Registered camera paths of four characterized sequences, one per capture geometry. Each point is a camera, colored from the first frame (purple) to the last (yellow), and the arrows show the viewing direction of every few cameras. Paths are drawn in the principal axes of their own reconstruction and scaled to the same length, so they compare in shape but not in size. (**a**) A BTG row pass keeps almost parallel views, with a 90th-percentile spread of 0.5° around the mean viewing direction. (**b**) A GrapeMOTS orbit, which covers an arc around one plant, converges its views on that plant, with a spread of 28.7°. (**c**) The 45° Botrytis flight covers a grid of parallel lines, with a spread of 48.9°. (**d**) The BLT side camera looks perpendicular to the motion of the robot, at 87° to the direction of travel, with a spread of 1.9°. Figure A4 shows the paths of all 29 sequences with a registered model, the 27 reconstructed sequences of Table 3 and the BLT side passes of 13 July and 15 September 2022, whose largest models hold 10 and 57 frames, below the gate.

Figure 2 places every characterized sequence on the measured quantities of Table A3 and Table A4, and three properties of the record stand out. The first is concentration, since six of the ten datasets were captured on one estate in Tomiño, four of them on the same 1.06 ha block and the other two on that block and a neighboring one. As a result, a method validated on these six datasets may have seen only one vineyard, one cultivar, and one trellis system under different sensors. The second is that registration is almost never partial. Every UAV video sequence and the GrapeSLAM video register in full, the four stills sets register at most 5% of their images, and only BLT occupies the middle ground, where the registered share depends on the session rather than on the dataset. The third is that parallax and viewpoint coverage separate the datasets far more finely than registration does. The Botrytis flights reach triangulation angles of 20 to 30° and a coverage close to 2 sr, while the other captures stay below 0.8 sr and, apart from one BTG part at 21°, at or below 12°, a difference that reflects their multi-angle off-nadir flight plan rather than the sensor. Figure 3 shows these capture geometries as registered camera paths. Resolution spans a factor of forty, from 0.3 Mpx to 12 Mpx, yet the requirement sweeps of Section 4.5 show it to be the least binding of these properties. Similarly, the frame-level quantities are close to zero almost everywhere, so blur, exposure, and static content do not limit reconstruction in this record. The exceptions are one Greek BLT pass whose side camera sees a third of static sky; the UK BLT pass with 12% of its pixels clipped; the GrapeCS-ML photographs, half of which fall below the sharpness threshold; and the WGISD photographs, 16% of which do. Finally, access is less uniform than the CC BY labels of Table 1 suggest, because WGISD is licensed for non-commercial use only, GrapeCS-ML states no license, and BLT is available only after registration.

### 3.6. Where and When

**Figure 4:**
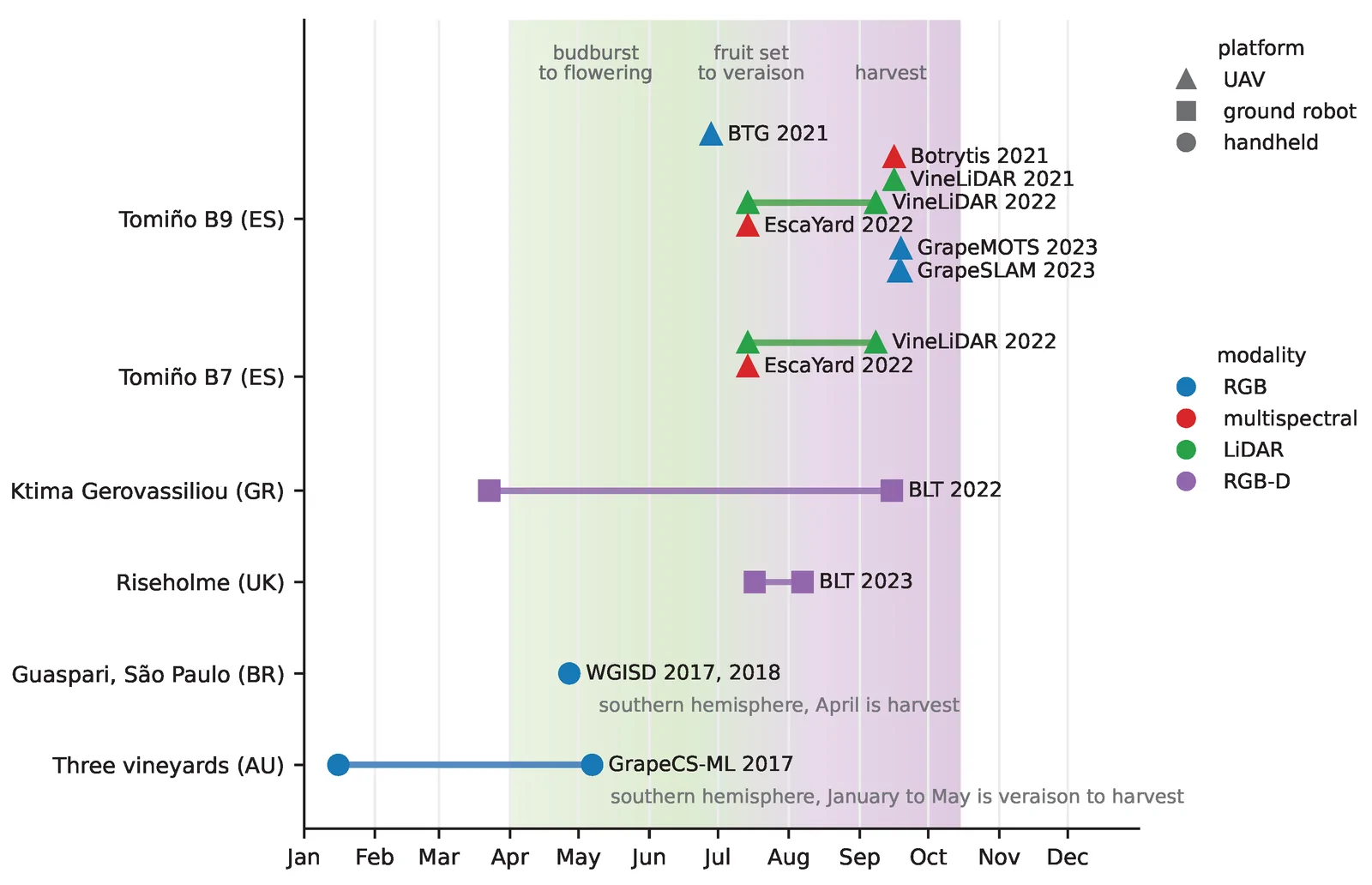
Where and when the characterized datasets were captured. Sites are listed vertically and the calendar month horizontally, with the marker showing the platform and the color the modality. The background follows the vine through the northern-hemisphere season, from the young green of budburst and flowering through the color change of veraison to the ripe violet of harvest, and the two southern-hemisphere captures are annotated with their local stage.

Figure 4 places every dated capture on a calendar. Read together with its site axis, it shows that the part of the record usable for reconstruction consists of one Spanish estate observed repeatedly from the air, one Greek vineyard observed from the ground through a season, and a handful of single-day captures elsewhere. Ten of the thirteen dated captures fall between fruit set and harvest, when the canopy is full and the fruit is visible. As a result, the early season, when the trellis structure is exposed, is represented only by the first BLT sessions. The shared estate is also an opportunity that none of the datasets exploits. The Botrytis flights and the first VineLiDAR scan were flown over the same block on the same day, 16 September 2021, and would provide a multispectral and a LiDAR view of one canopy state if the LiDAR files from that day carried a coordinate reference system (CRS). One dataset, GrapeSet, states no acquisition date at all, and for a record whose main gap is temporal, that omission is itself a finding.

The ten characterized datasets, with the thirty-four sequences whose measurements Table A3 and Table A4 summarize, are the population on which every experiment of Section 4 is run.

## 4. Through the Reconstruction Lens

### 4.1. The Fixed Instrument

To establish whether the characterized datasets can be reconstructed at all, and how well, we process every sequence that passed the registration gate with one fixed pipeline, so that the rows of Table 3 differ only in their input. The frames are the same lists that were sampled for the characterization, at most 200 per sequence at no more than two per second and resized to a width of at most 1600 pixels. Their poses and sparse points come from the same SfM configuration, after which the images and the camera model are undistorted so that the trainer sees a pinhole camera. The radiance field is optimized with the gsplat [59] implementation of 3DGS for 30,000 iterations from the sparse points. The initial Gaussian size is the nearest-neighbor distance, color is plain RGB without spherical harmonics, and densification follows the default schedule, which makes this a minimal rather than a tuned trainer. Novel-view quality is reported as peak signal-to-noise ratio (PSNR) [21,22], structural similarity index measure (SSIM) [60] and learned perceptual image patch similarity (LPIPS) [61] with the AlexNet backbone, computed on held-out views that are chosen once per sequence and never seen in training. The usual rule of holding out every eighth frame leaves each test view with training views half a second away on both sides, so that novel-view synthesis becomes interpolation between near-duplicates. Every sequence is therefore trained a second time with contiguous blocks of four frames held out, which places the middle views of each block two sampled frames from the nearest training view rather than one, and both results are reported side by side. The scores cover registered frames only, and the spread given with each mean is the spread across held-out views rather than across runs. A repeated training of the same sequence with another seed moves the mean by 0.3 dB in the median and by up to 0.9 dB on the UAV sequences and 1.5 dB on the BLT front passes, so that differences below about 1 dB between two rows are not evidence, and the ablations of Section 5 average three seeds. The complete configuration, including every hyperparameter that differs from the gsplat defaults, is given in Appendix A, and Table A1 lists it for every reconstruction.

### 4.2. Registration Across the Record

The registered share of every characterized sequence is given in Table A4 and in the third row of Figure 2, and the first result of the task is that registration is decided by the capture rather than by the dataset. Every UAV video sequence, whether a row pass or an orbit, every Botrytis flight, the GrapeSLAM video and the WGISD video frames register in full or nearly so. The four stills sets register at most 5% of their images, in two-image models, which is what their missing overlap looks like when it is measured. Between these two outcomes lies only BLT. Its side-camera passes register from 1 to 100% depending on the session, and two of its sessions are examined separately in Section 5.3. Because the solver’s matching and initialization are multithreaded, two runs on identical input need not give the same result, and when the fixed configuration is run five times on the same frames, as reported in full with the BLT mitigation below, the untreated front-camera passes register in zero to two runs out of five, so that a single run of such a pass is one draw rather than a result. On the UK front pass, a likely mechanism is the initialization, because COLMAP chooses its initial pair by the number of matches, which on a camera moving along its own viewing direction favors adjacent frames with little parallax. A sideways camera on the same robot sees the row pass by with real parallax between neighbors and registers in four runs of five, while the two UAV sequences used as controls register in three runs of three, and the same row pass sampled at half a frame per second registers in none, which fixes the spacing floor reported in Section 4.5. Therefore registration stability, reported as the success rate over five runs, is a property that a single run cannot reveal, and it is reported wherever a sequence registered unreliably. Mean reprojection error lies between 0.08 and 0.86 pixels over all 34 registrations, the stills sets included, and between 0.29 and 0.86 pixels for the 27 reconstructions (Table A1), so it does not separate the datasets.

### 4.3. Reconstruction Quality

**Figure 5:**
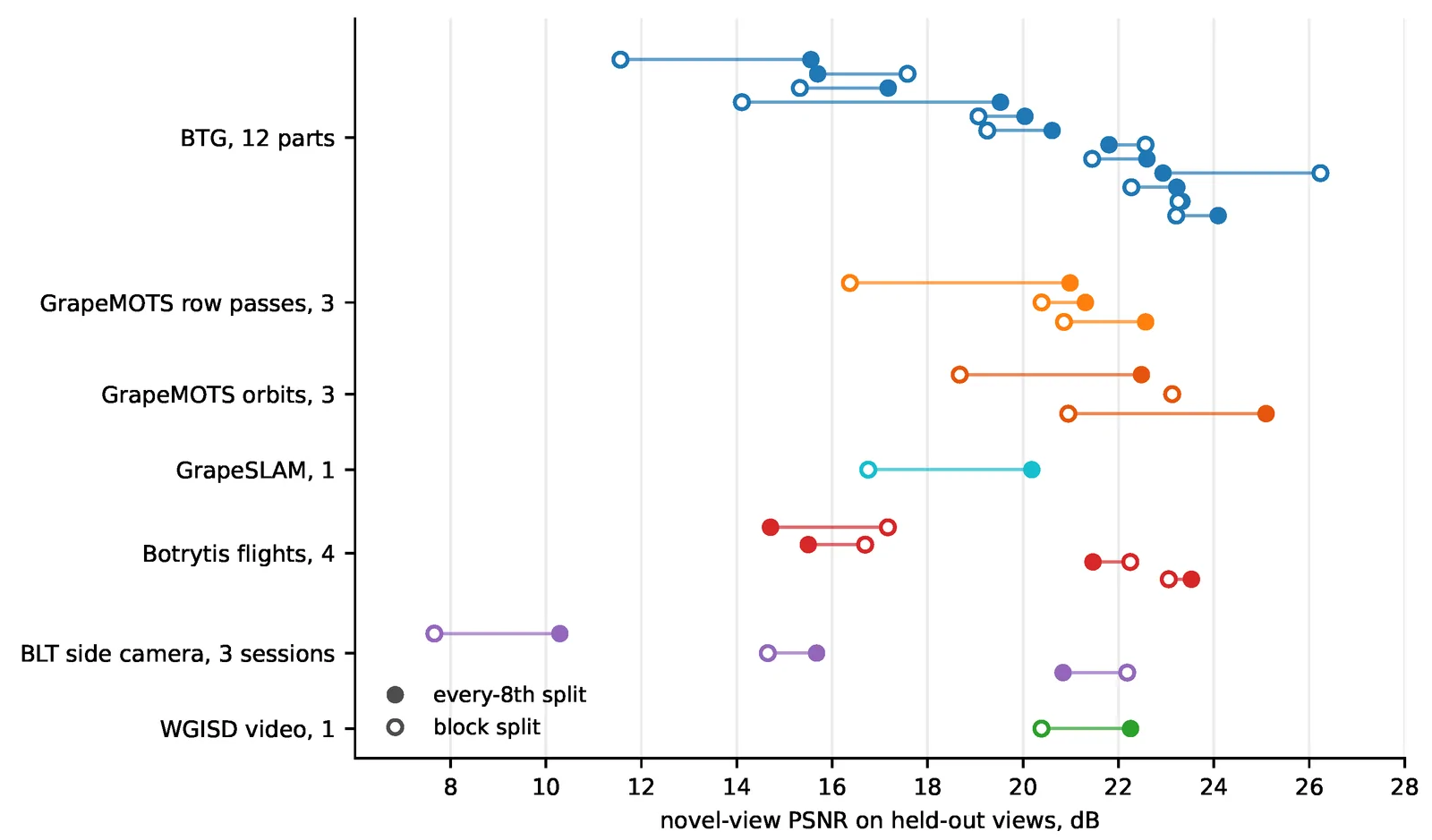
Novel-view PSNR of every reconstructed sequence on both held-out splits, grouped by dataset. Each sequence is a pair of markers joined by a line, filled for the every-eighth split and open for the block split, so that the spread of markers within a row shows how much quality varies inside one dataset and the length of each line shows how much of a sequence’s score the standard split owed to interpolation between neighboring views. Lines pointing to the right mark the sequences that scored higher on the block split.

**Figure 6:**
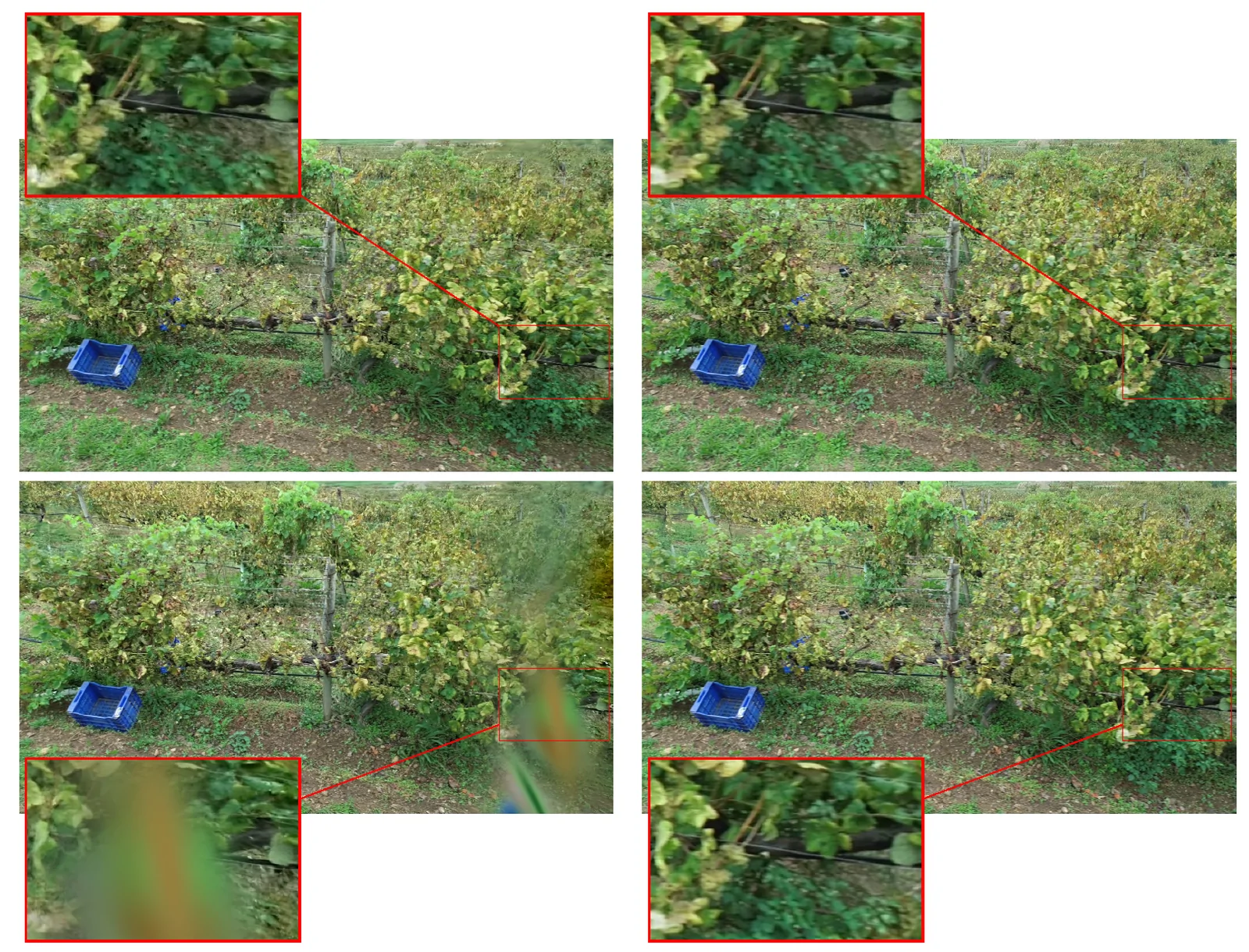
A single held-out view rendered by the models of the two held-out splits, with each render on the left and the ground-truth frame on the right. Top: model trained on the every-eighth split (25.6 dB). Bottom: model trained on the block split, for which this view is the first frame of a held-out block of four (21.6 dB). The red insets magnify the right edge of the frame. The blurred, discolored streak in the bottom-left inset is the floating artifact that the block-split model renders there, because it has seen no neighboring view, and the every-eighth model avoids it. The view is frame 16 of GrapeMOTS orbit 8.

**Figure 7:**
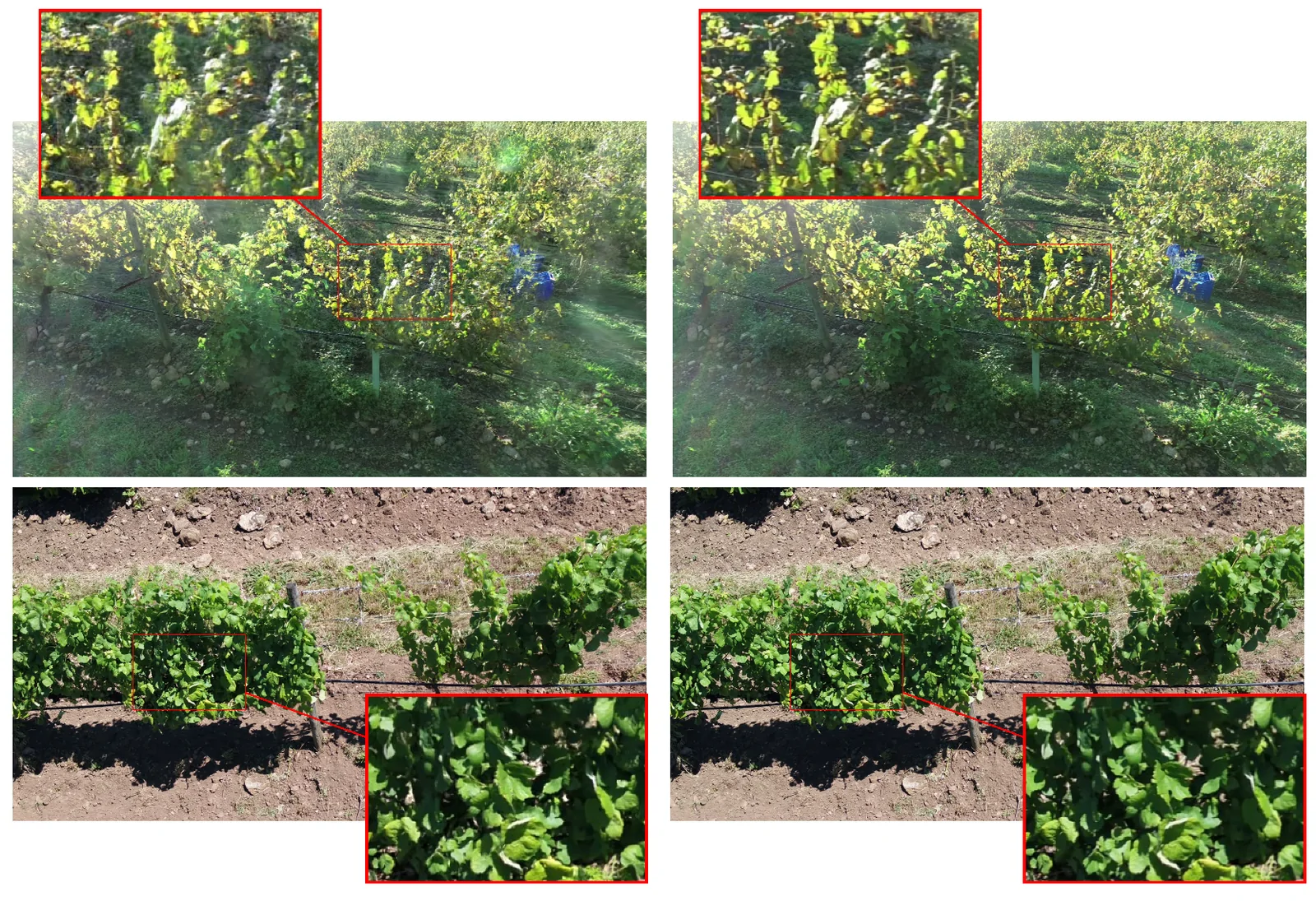
Median held-out views of two reconstructions on the every-eighth split, rendered view on the left and ground-truth frame on the right, with the red insets magnifying the same canopy region in both. The views shown are those whose PSNR is the median of their sequence, not the best, so that they represent the row of Table 3 they belong to. Top: GrapeMOTS row pass 1, frame 16 (21.5 dB). Bottom: BTG row 7.2, part 3, frame 40 (25.2 dB).

Table 3 lists the 27 sequences that passed the gate, and Figure 5 shows the same rows on both splits, grouped by dataset, which makes two properties visible that the table hides among its columns. Three Greek BLT side passes, of 23 March, 13 July and 15 September 2022, did not pass the gate in the single characterization run and are not reconstructed, although the 23 March pass registers in four of five repeated runs (Section 5.3), so its absence reflects one failed draw rather than an unregistrable pass. The quality of the untreated reconstructions ranges from 10 to 25 dB on the every-eighth split, and the range within a dataset is as wide as the range between datasets. The twelve BTG parts span 15.6 to 24.1 dB although they come from one flight over one block. The four Botrytis flights, reconstructed from their aligned color composites, span 14.7 to 23.5 dB with the 45° flight best and the afternoon nadir flight worst. The BLT side passes span 10.3 to 20.8 dB across the season. Consequently, a single sequence cannot represent a dataset, and the spread within a dataset is itself a result of the characterization. Above the registration floor, parallax does not predict quality, since the Botrytis flights at 20 to 30° of triangulation angle score no better than the BTG parts at 6°, whereas the distance between the two splits, the length of each line in Figure 5, does. The block split lowers the score by more than 1 dB on 13 of the 27 rows, by up to 5.4 dB, and by more than 3 dB on two of the three GrapeMOTS orbits, the backlit row pass, the GrapeSLAM video and two BTG parts, which is the share of the every-eighth score that interpolation between near-duplicate views contributed (see Figure 6). Five rows score more than 1 dB higher on the block split, by up to 3.3 dB, namely two BTG parts, two Botrytis flights, and one BLT session. For the two BTG parts, which have four to six held-out views, this follows from the every-eighth split always including the first frame of the recording, which the training views cover from one side only, so that on a short part that single view weighs heavily on the mean. The remaining nine rows change by less than the 1 dB that separates two training runs. Figure 7 shows the median held-out view of two sequences rather than their best. For GrapeMOTS, whose annotations give a grape mask on every frame, the held-out views can also be scored inside and outside the clusters, and on the three row passes, the clusters score 2.1 to 4.4 dB higher in PSNR and higher in SSIM than the surrounding canopy on both splits, which confirms that wind-moved leaves rather than fruit carry most of the error. LPIPS separates the two regions only on the every-eighth split, where it rates the clusters better on row passes 2 and 3 and slightly worse on the backlit row pass 1.

### 4.4. Scale Anchors

SfM recovers geometry up to a similarity, so the scale and shape of a reconstruction can be validated only against an external reference, and the scale-anchor column of the readiness table in Section 6 lists what each dataset provides. Where a trajectory is available, the registered camera positions are aligned to it by a similarity transform and the root mean square distance after alignment is reported. The BLT passes align to the robot’s RTK-GNSS fixes with 7 to 10 cm of error over 52 to 141 cameras, and the GrapeSLAM video aligns to its flight log with 5.6 cm over 49 of 52 cameras, which places the scale of these reconstructions within the reference’s own accuracy. The Botrytis flights align to the EXIF GPS of their captures with about 1 to 1.4 m of error, which is the error of consumer GPS rather than of the reconstruction, and their seven surveyed GCPs cannot be identified in imagery taken from 20 m above ground. The remaining datasets provide no anchor for an image reconstruction: BTG and GrapeMOTS state an RTK platform but deposit no trajectory, VineLiDAR carries a coordinate system in eight of its ten files but no imagery, and the three stills sets ship no position. Therefore, for five of the ten datasets, the scale of a reconstruction can be set only from a known object in the scene, VineLiDAR’s coordinate system anchors point clouds but no imagery, and for two of the others, the deposited reference is weaker than its documentation claims.

### 4.5. Requirement Sweeps

**Figure A1:**
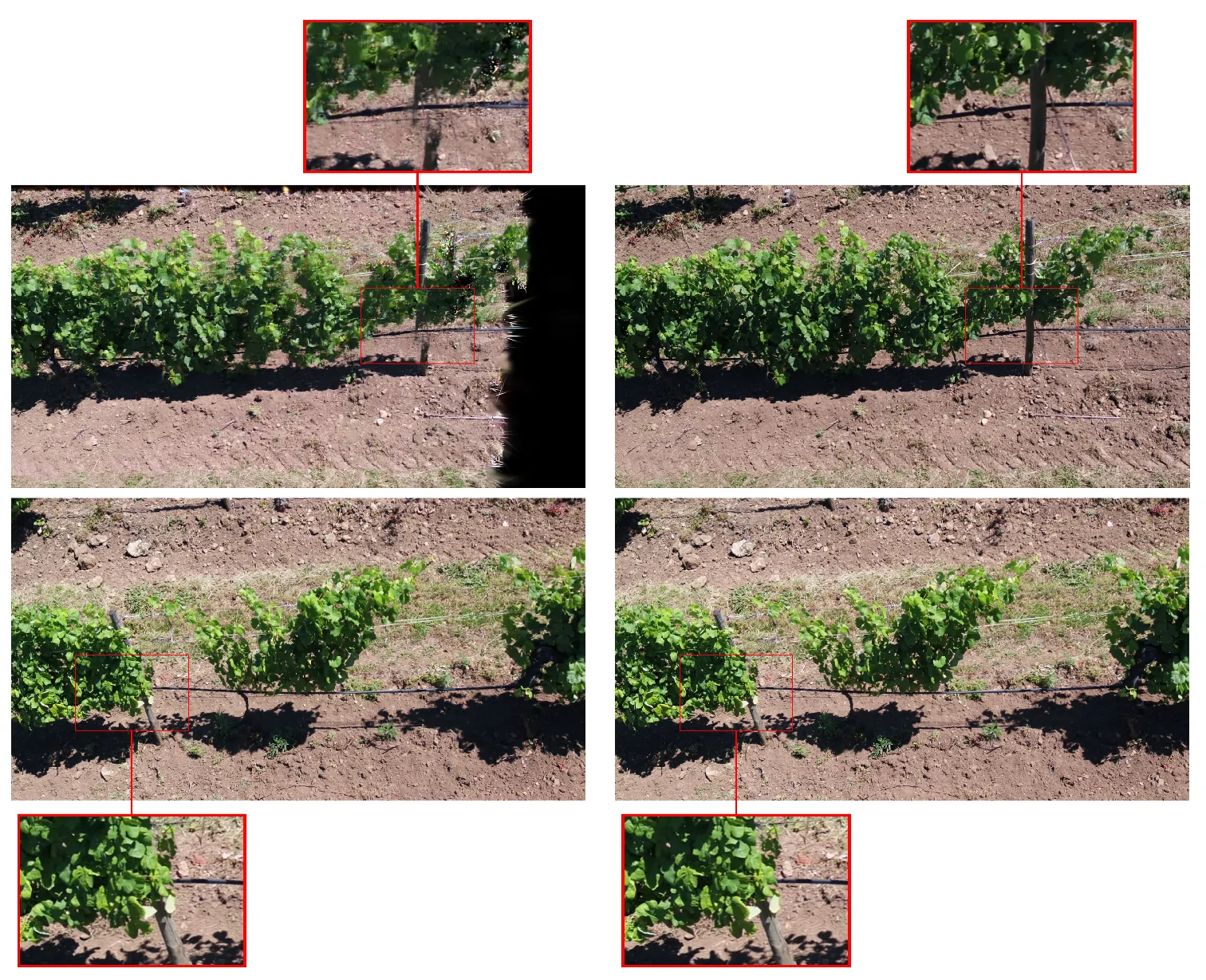
Median held-out views of BTG row 7.2, part 3, at the two extremes of the frame-spacing sweep, with the rendered view on the left and the ground-truth frame on the right, with the red insets magnifying the same region in each pair. The black region in the top render is part of the scene that the sparser training views do not cover, and the top inset shows a trellis post that is missing from the sparse render. (**Top**): frames sampled at 0.5 per second (12.9 dB). (**Bottom**): frames sampled at 4 per second (28.5 dB).

To turn the measured properties into requirements, three of them were varied one at a time on two UAV row passes while everything else stayed at the protocol value (Table A2 in Appendix A). The two passes are the backlit GrapeMOTS pass, because it is the hardest registered sequence, and a BTG part, because it is a dense canopy under good light. Frame spacing is the binding property among the three that were varied. Sampled at half a frame per second, which at these flight speeds places consecutive frames about two seconds apart, the backlit pass registers 2 of 15 frames and fails in all five repeated runs, while the BTG part still registers but falls to 14 dB. At one frame per second, the backlit pass registers 80% of its frames in two models. At the protocol’s two frames per second, both register in full, and at four, they gain a further 3 to 4 dB (see Figure A1). Therefore, a sequence sampled below about two frames per second, or equivalently with a spacing between consecutive frames above about 3% of the path, should not be expected to register under standard SfM. Image width is not binding within the range of widths that the record spans. When every reconstruction is scored at a common width of 640 pixels, training at 640 costs at most 1.1 dB against training at 960 or 1600, and training at the native 3840 or 4096 pixels loses 0.3 to 0.9 dB rather than gaining. Therefore, the 640 × 480 photographs of GrapeCS-ML would have been usable had they overlapped. Frame count shows no cliff either, since 15 frames spanning 7 seconds register and score within 1 to 2 dB of sixty.

Three findings follow from the reconstructions and the sweeps reported in this section. Registration followed the capture rather than the dataset, since every video registered in full or nearly so, no stills set did, and the one dataset between the two outcomes, BLT, differed by session and by run rather than as a whole. Novel-view quality varied as much within a dataset as between datasets, with ranges of nearly 9 dB inside a single flight over one block. Frame spacing was the one capture property that limited reconstruction, since registration failed once consecutive frames lay more than about 3% of the camera path apart, whereas image width and frame count left registration unchanged across the range that the record spans.

## 5. Failure Cases and Mitigations

Three of the characterized datasets fail or degrade under the fixed configuration for a reason that lies in the capture rather than in the scene, and for each, a preprocessing step suggests itself from a visual inspection of the frames. Each step was tested as an ablation under the same instrument as everything else, with registration reported first because it does not depend on the treated images, and with novel-view quality reported against each condition’s own treated frames, so that a treatment is judged on whether the reconstruction becomes more consistent rather than on whether its colors match an untreated reference. Band alignment restored registration of the multispectral composites, while dehazing a backlit pass raised PSNR only inside the fruit regions, on one seed and without any gain in SSIM or LPIPS, which this study cannot separate from noise. Masking the static chassis of a ground robot did not consistently improve the registration of its forward-facing camera, and removing a standing tail that a cutting fault had left in two of its passes reduced the share of runs that registered.

### 5.1. Multispectral Band Composition

**Figure 8:**
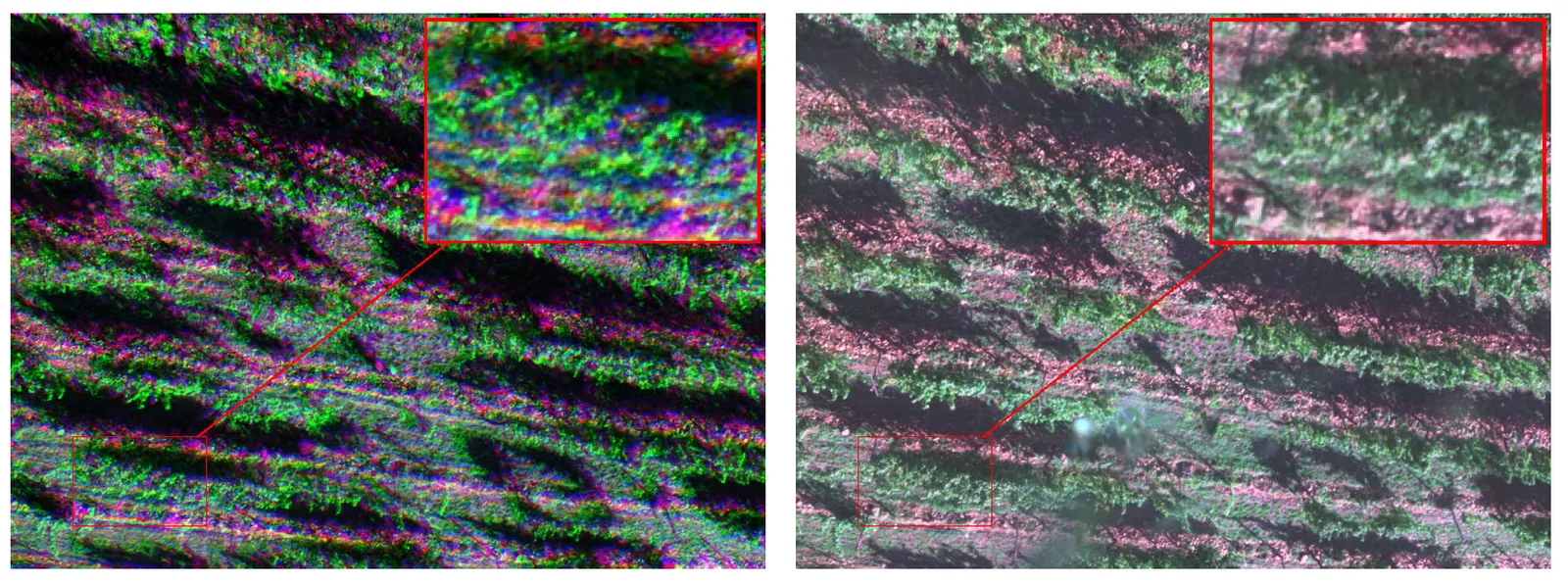
The same held-out view of the 45° Botrytis flight rendered from the naive composite of the blue, green, and red bands (**left**, 17.1 dB) and from the aligned and jointly stretched composite (**right**, 24.3 dB). The red insets magnify the same canopy row, where the naive composite splits every row edge into colored fringes and the aligned composite keeps leaves and soil apart. Each render is scored against its own composite, and the naive render is shown with the same 30-pixel border removed as the treated one. The view is the median held-out frame of the treated condition, and Table 4 reports the full ablation.

The Botrytis flights record five bands through five lenses, so that stacking the blue, green and red bands as recorded leaves a parallax offset between them that every edge in the composite inherits. Table 4 isolates what each step of the correction does to registration and to quality, and Figure 8 shows the same held-out view before and after it. Alignment is the step that matters for geometry: the 45° flight registers 109 of 200 frames in two models from the naive composite and all 200 in one model once the bands are warped onto the green frame, and radiometric correction without alignment leaves registration where it was. One homography per band, estimated as the median over sample captures, is both sufficient and better than a homography per frame, which fails to converge on 14% of the frames of the 45° flight and 37% of the nadir flight and scores 1 to 5 dB lower. The remaining steps act on quality rather than on registration: stretching the three bands jointly scores about 1 dB above stretching them independently, and the 30 pixel border crop is worth 3 to 4 dB, because the warp leaves an inconsistent margin that the optimizer would otherwise have to explain. However, the green band alone scores higher than any composite, 25.9 against 23.4 dB on the 45° flight, so the composition buys a color image at a cost in consistency, and a false-color composite of the three non-visible bands registers as fully as the visible one and reconstructs within about 2 dB of it, which answers whether the agronomically relevant bands are lost to the treatment: they need not be. The composites are normalized by a joint percentile stretch and a gray-world white balance rather than calibrated to reflectance with the deposited panel captures, so they serve visual reconstruction rather than phenotypic analysis, which would need that calibration and all five bands carried by each Gaussian.

### 5.2. Backlit Capture and Lens Flare

**Figure 9:**
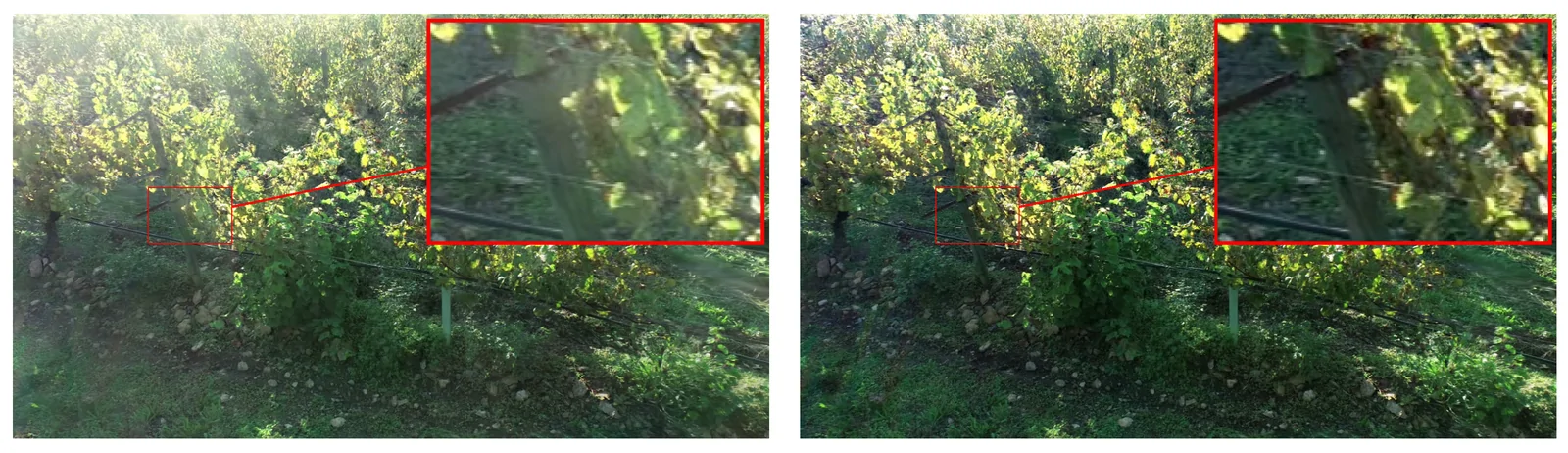
The median held-out view of the backlit GrapeMOTS row pass rendered from the frames as recorded (**left**, 21.4 dB) and after dehazing with the lens ghost masked (**right**, 20.3 dB). The red insets magnify the region of one annotated cluster, which is partly hidden by leaves and gains contrast after the treatment. Over the full frame the treated render scores lower, whereas inside the dataset’s grape-cluster masks the treatment raises the score. Table 5 reports the full ablation, including the scores inside and outside the cluster masks.

The backlit GrapeMOTS pass registers in full in every condition, so its treatment is a question of quality alone, and Table 5 gives the answer at two scales. Over the full frame, the pass as recorded scores as well as or better than any treatment, and the control pass flown under even light is indifferent to all of them, so dehazing does not improve the frame as a whole. Inside the grape-cluster masks that the dataset ships, dehazing raises the score of the first seed by 0.7 to 1.5 dB while the surrounding canopy moves the other way, which is the one place where the treatment may help. SSIM and LPIPS inside the clusters, however, do not improve in any dehazing condition, so the gain lies in pixel values rather than in structure or perceptual similarity. Because these region scores come from one seed, the size of the gain is less certain than its direction, which all four dehazing conditions share. The gain of the full method, 0.7 dB, is below the 1 dB that Section 4.1 sets as the threshold for evidence, so dehazing is reported here as unresolved rather than as a repair. The guided filter that refines the transmission map adds 0.3 dB on the clusters, which is less than the seed noise of Section 4.1, so its effect is not resolved. The estimate of atmospheric light from the mean of the brightest pixels, which guards against clipped sensor values, scores below the single brightest pixel, and the lens-ghost mask alone lowers the cluster score, so that only its combination with dehazing matches dehazing alone. Figure 9 shows the median held-out view as recorded and after dehazing with the ghost masked.

### 5.3. Static Chassis Content and a Standing Tail

**Figure A2:**
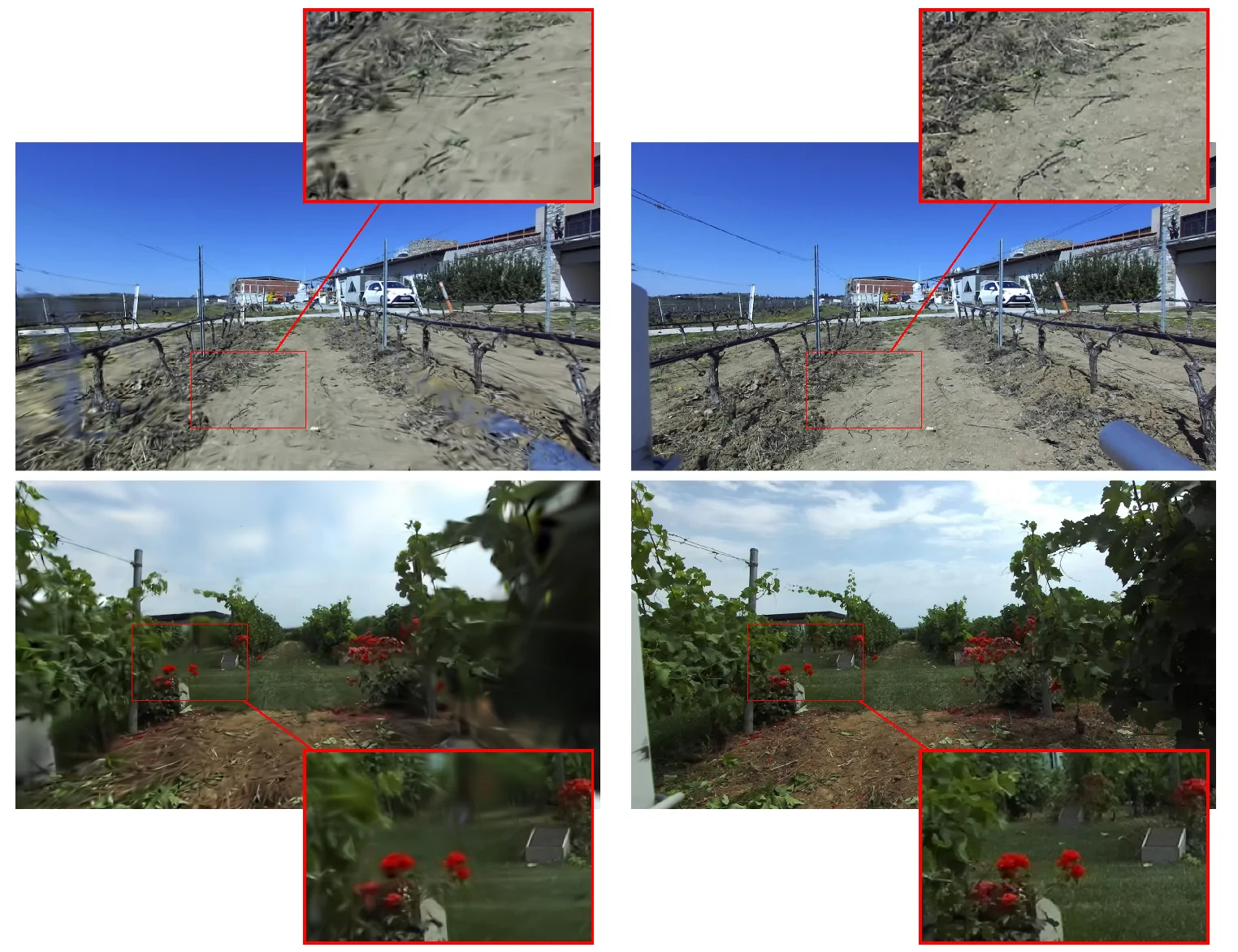
Median held-out views of two BLT front-camera passes in Greece, rendered view on the left and ground-truth frame on the right, with the red insets magnifying the trellis wires in March and the canopy in June. (**Top**): 23 March 2022, with the bare trellis before budburst (22.8 dB). (**Bottom**): 8 June 2022, with a full canopy in the near field of view (17.7 dB).

The run records are consistent with this result. COLMAP starts from the pair of frames with the most verified matches, and with the tail present, this pair is a standing frame and a frame taken a few steps before the stop, which share many matches across a real baseline. Without the tail, the pairs it tries between moving frames share a median of 32 verified matches, and the failing runs stop after 59 to 115 attempted pairs with two or three registered frames. This suggests that a camera that looks along its direction of travel finds too few matches between neighboring frames, which would also explain why the UK front pass, which has no standing frames, fails in three runs of five. The side camera of the same robot sees the row pass by and registered in four runs of five on the one session repeated. The three Greek front passes that registered as single runs reconstruct at 21.9, 18.5 and 16.3 dB from March to June, a decline with the growing canopy that the side camera also shows (see Figure A2).

**Figure 10:**
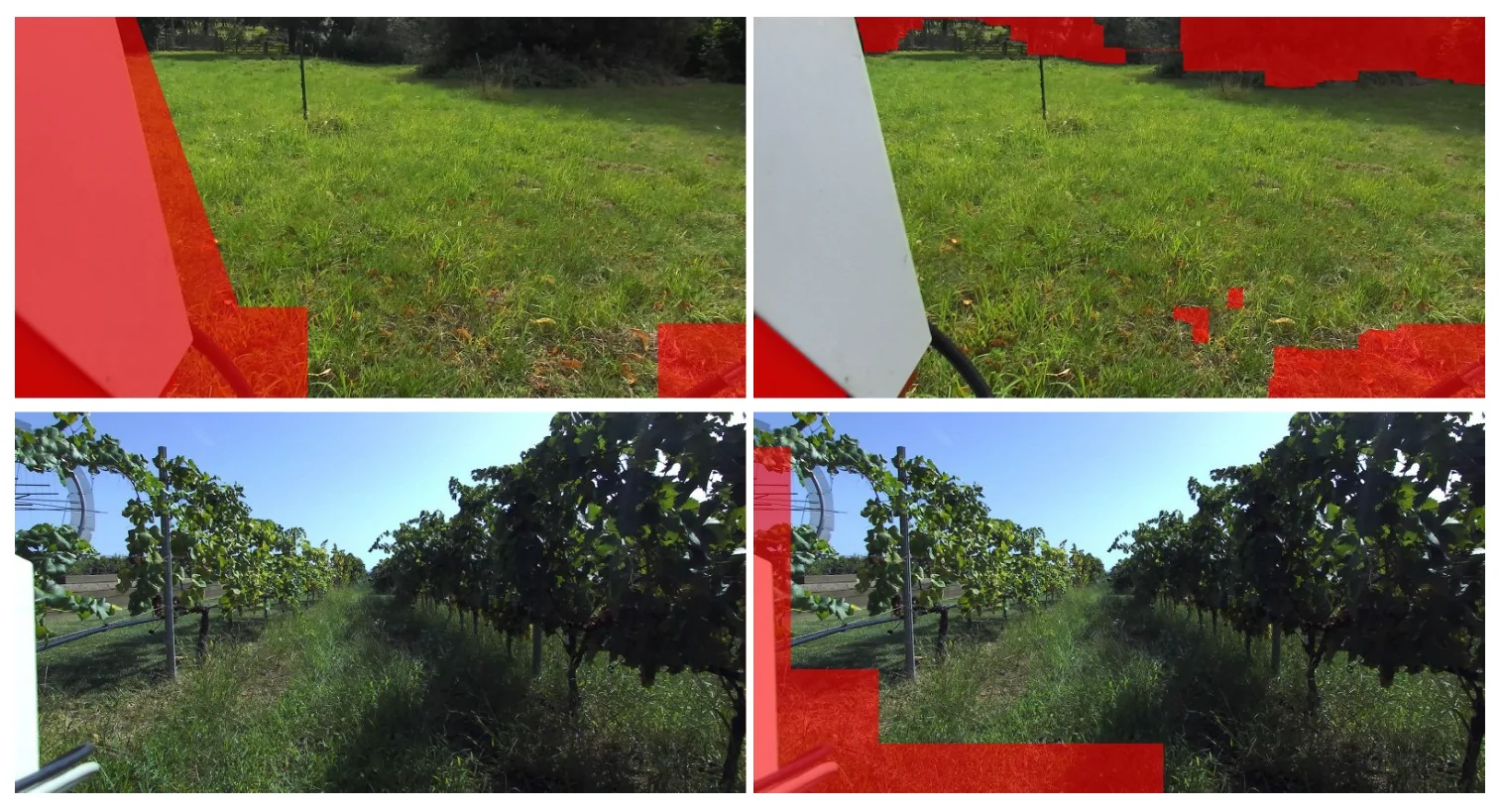
The chassis masks of Table 6 drawn in red over one frame of each front camera. (**Top left**): the UK pass with the fixed-region mask, covering 28.8% of the frame. (**Top right**): the same frame with the color-threshold mask, 18.8%, which also removes part of the tree line. (**Bottom left**): a frame of a Greek pass as recorded, without any mask. (**Bottom right**): the same frame with the hand-drawn mask, 12.5%, which covers the chassis edge, the wheel and the lower border. The masked region is excluded from feature extraction and from the training loss.

**Figure A3:**
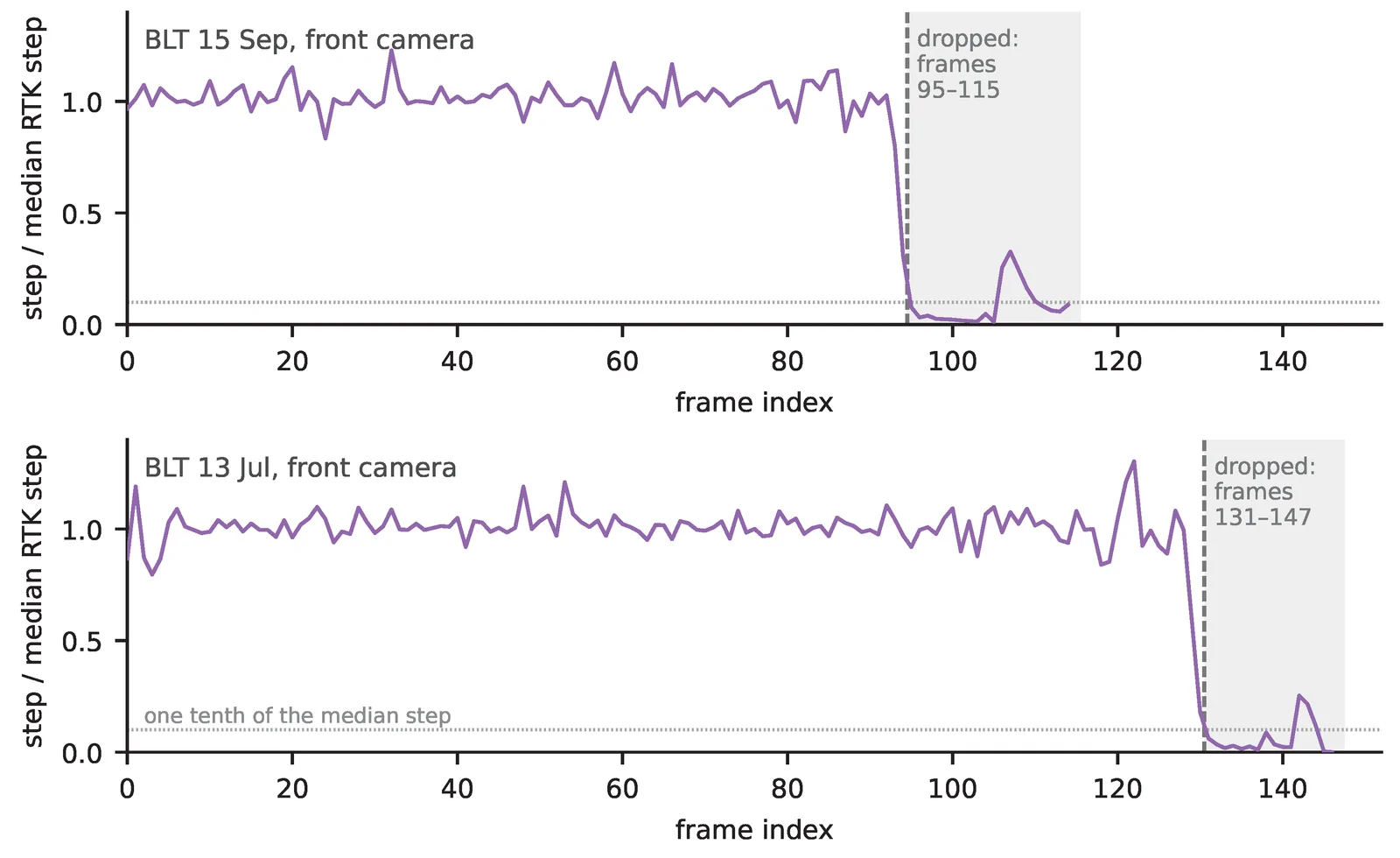
Step between consecutive frames of the BLT front camera as a ratio to the median RTK step, with frames 0.47 s apart. A value near one is a normal move between frames, and a value near zero means the robot barely moved. The shaded frames at the end of each pass, where the step falls below a tenth of the median, are a standing tail: the robot had stopped at the row end while frames kept recording, because the original cut selected frames on the time each message was written to the recording, and the odometry was written 7 and 12 s after the images. These motionless frames are what the trimmed condition of Table 6 removes. (**Top**): 15 September 2022. (**Bottom**): 13 July 2022.

The front camera of the BLT robot carries two faults that a single run cannot separate. The first is static content, because the chassis occupies a fixed region of every frame and its features match across the whole sequence (Figure 10). The second is a cutting fault in two of the Greek passes. On 13 July and 15 September 2022, the passes were selected on the time at which each message was written to the recording, and because odometry was written 7 and 12 s after the images it describes, the two passes end with 17 and 21 frames of the robot standing at the row end (Figure A3), and on 15 September, the first 20 s of the row are missing. We re-cut both passes on the time stamps that the images carry, and the corrected cut is the one reported everywhere else in this paper.

**Figure 11:**
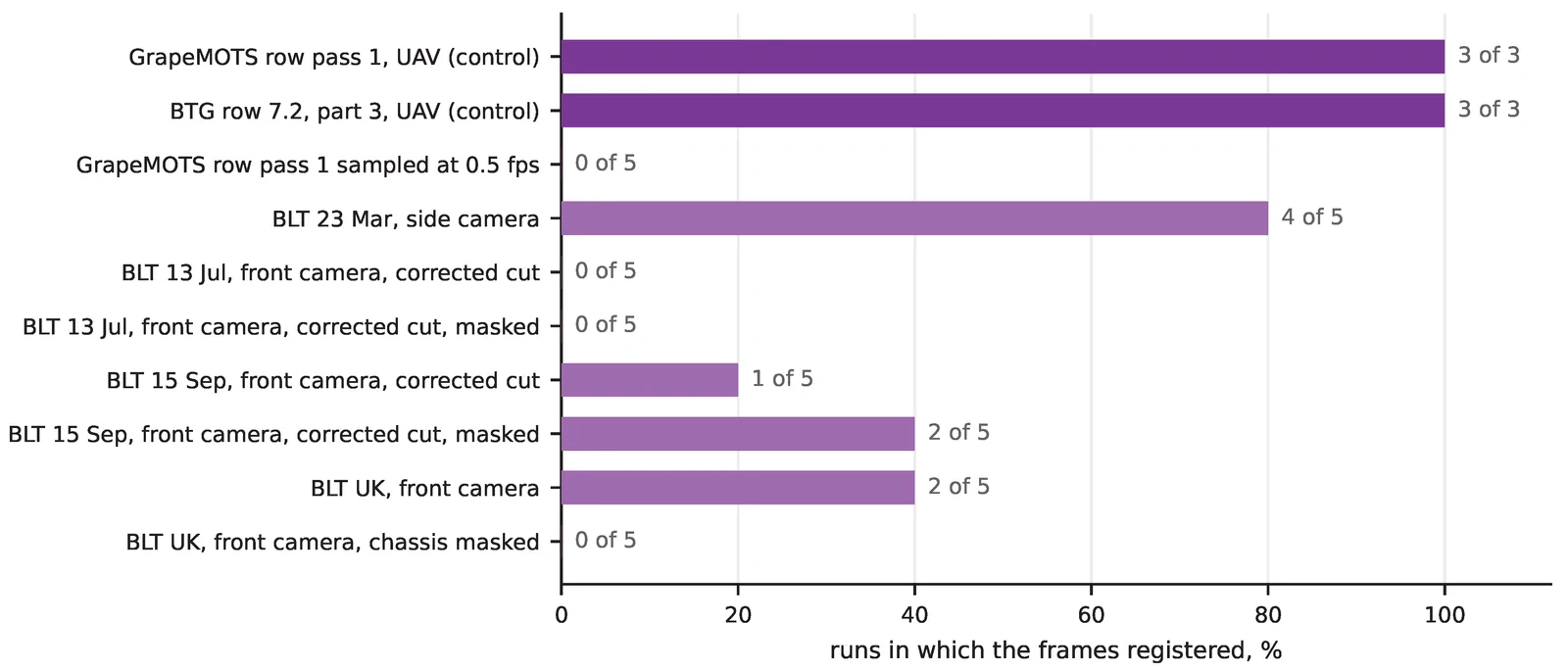
Registration success over repeated runs of the fixed configuration on the same frames. Each input is run repeatedly, five times here and three for the two UAV controls, and a run counts as registered when its largest model holds at least half of the frames, so each bar shows how many of the repeated runs succeeded. Each bar carries the color of its dataset’s readiness class in Figure 1, dark purple for ripe and light purple for ripening. The two UAV passes register every time, the pass sampled at half a frame per second never does, and the BLT front camera in only zero to two runs of five. The 13 July and 15 September passes are shown on the corrected cut of Section 5.3. Because the same frames in the same configuration register on one run and fail on the next, registration is not deterministic, and an unstable capture must be repeated before its reconstruction is reported.

Table 6 separates the two faults with five registration runs per condition, and Figure 11 compares the corrected passes with the other inputs that were registered five times. Neither treatment restores registration. Masking the chassis raised the original 13 July pass from one to four runs of five, a difference that is not significant (Fisher exact test, p=0.21) and moved every other pass and cut by at most two runs in either direction (p≥0.44). Removing the standing tail lowered success instead of raising it, from 9 of 20 runs across the four original-cut conditions to 2 of 20 after trimming (stratified exact test, p=0.035) and 3 of 20 on the corrected cut, although the trimmed passes are also 17 to 21 frames shorter. Where a run registered, the tail did not change image quality, which reached 16.6 dB trimmed against 16.7 dB untrimmed on the same twelve held-out views.

### 5.4. Boundaries of the Mitigations

Each mitigation has a domain outside which it is unnecessary or ineffective. Band alignment is required whenever a multi-lens multispectral sensor is composed into a color image and is unnecessary when a single band serves the purpose, which for reconstruction it does. Dehazing does nothing for the frame as a whole or under even light, and on the fruit of a backlit pass, it raises PSNR without improving SSIM or LPIPS, so a backlit capture is better avoided than repaired. Chassis masking removes static content but has no consistent effect on the registration of the BLT front camera, with or without the standing tail, most likely because a camera facing its direction of travel finds too few matches between neighboring frames.

## 6. Readiness and Gaps of the Record

### 6.1. Readiness Classes

**Figure 12:**
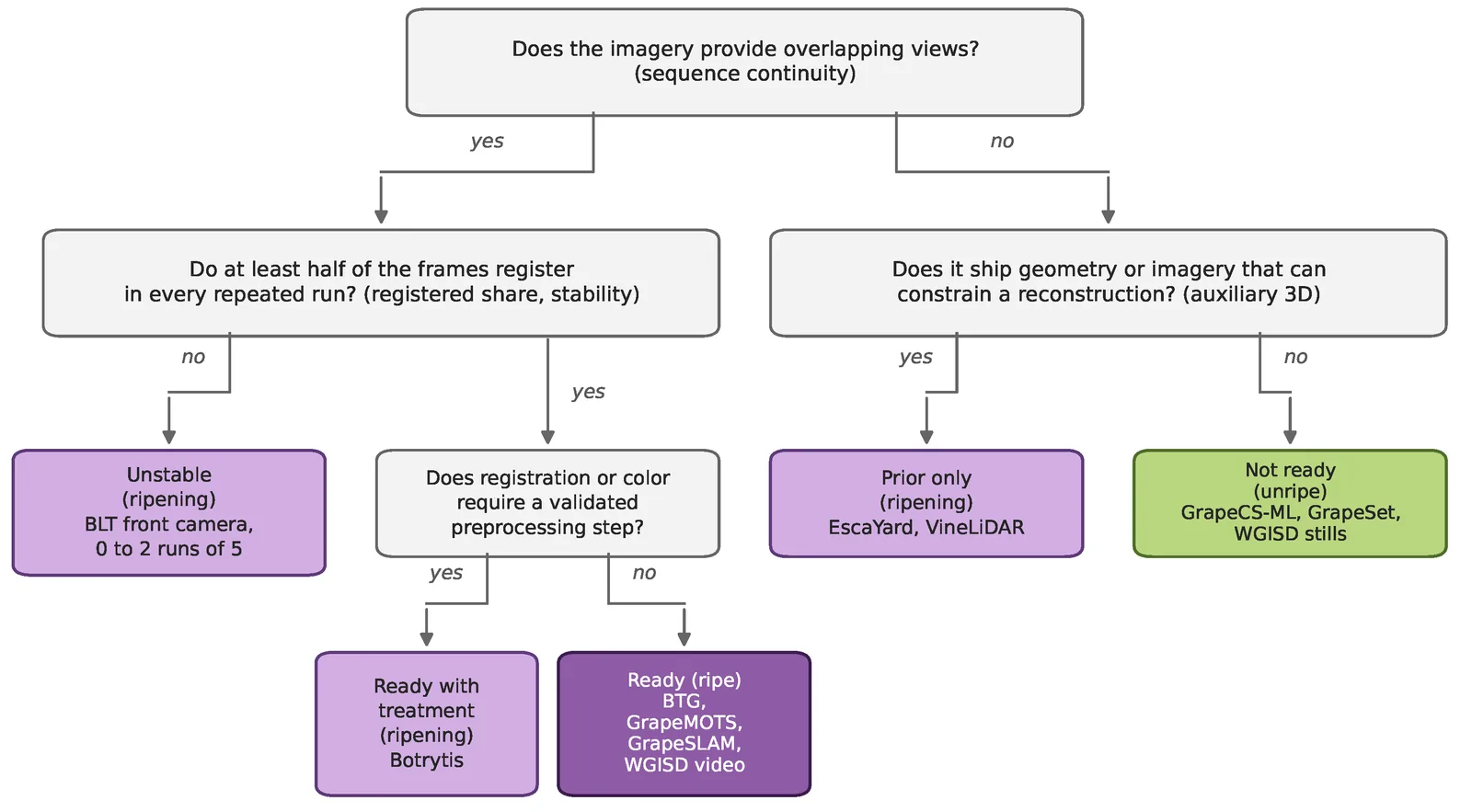
Decision procedure that assigns a readiness class from measured properties. To classify a new dataset, start at the top question and follow its measured values down to a leaf. Each question is answered from a property of Table A3 and Table A4 under the fixed configuration, and each leaf names the class, the color it carries in Figure 1, and the characterized datasets that reach it. The procedure is applied per capture where a dataset has more than one, which is why WGISD’s video frames and its photographs reach different leaves.

The measurements of Section 3, Section 4 and Section 5 combine into one verdict per dataset, and three classes summarize it, each with a test that can be applied under the fixed instrument. A dataset is ripe when its sequences register in every run made of them and reconstruct without any treatment. Every sequence of the three ripe datasets was registered at least twice (Table 7), so a ripe verdict states that no run has failed rather than that failure has been ruled out. A dataset is ripening when it yields a reconstruction, or a constraint on one, only at a stated cost, which may be a preprocessing step, repeated runs to obtain a registration, or use as a geometric prior rather than as imagery. A dataset is unripe when its imagery fails under the fixed instrument whatever preprocessing is applied, because it provides no overlapping views. Within the ripening class, ready with treatment means that registration or a usable color image requires one of the preprocessing steps of Section 5; unstable means that the same input registers in some runs and not in others; and prior only means that the dataset can constrain a reconstruction but cannot initialize one. Table 7 gives the verdict for each characterized dataset with the measured values and the evidence on which it rests, and Figure 12 turns the same tests into a procedure for a dataset outside the record.

### 6.2. What the Record Lacks

Read column by column, Table A3 and Table A4 show not only what the ten datasets contain but which combinations of properties no dataset in the record has, and Table 8 lists the absences that matter for reconstruction, each with what a dataset filling it would make possible and what it would cost beyond the data that already exist, in two groups. The first group needs new recordings, since no UAV capture returns to the same block in more than one season, no ground-level capture exists outside two sites, and no UAV capture shows the dormant season. The second group needs no recording at all, since the data exist and lack only a deposited trajectory, a coordinate system or one line of documentation. The record is also unevenly documented, and the documentation reliability row of Table A4 shows that for eight of the ten datasets, at least one documented value does not agree with the data, so that a dataset author’s single most useful addition is often not a new capture but a trajectory, a coordinate system or a corrected count.

### 6.3. Recommendations for Dataset Creators and Users

The measurements of Section 3, Section 4 and Section 5 translate into concrete recommendations, which Table 9 collects for the two audiences of this study. For dataset creators, they state what to record and what to document to make a new dataset reconstructable without the treatments of Section 5. For dataset users, they state what to check before reusing an existing dataset and how to evaluate a reconstruction before reporting it. Each recommendation rests on a result measured in this study rather than on general practice, and the table points to the section in which that result is reported.

### 6.4. The Online Catalog

The characterization is kept current online, so that the record can grow under the same measurement as the ten datasets reported here. The catalog is published at https://dragons-project.github.io/grapes-ready-for-picking/ (accessed on 31 August 2026). It presents the documented and measured values of every dataset, the readiness classes, the gaps, and the recommendations of this section, all generated from one table. A dataset that the search missed, or one published later, can be proposed through a submission form on the catalog page, which collects the deposit, the data paper, and the capture details and files them as a structured entry in the catalog repository. Proposals and corrections can also be sent by e-mail to the corresponding author. Before it enters the table, a proposed dataset is checked against the requirements of Section 3.2 and characterized with the same fixed configuration, schema, and repeated runs as the ten above. Its row is then comparable with theirs, and its class follows from Figure 12.

## 7. Limitations

Several limitations of this study follow from its design and should bound how its numbers are read. The characterized record is small and concentrated, with six of ten datasets from one estate, so that rows within BTG, GrapeMOTS and Botrytis share one flight, one plot or one block and are therefore not independent samples, and the per-dataset ranges of Table 3 describe those captures rather than the datasets’ sites. One SfM configuration and one minimal trainer were used throughout, because comparability across rows was the goal, and a tuned pipeline, a different initialization or a solver that chooses its initial pair by parallax would move every absolute number and might register the BLT front passes that this instrument registers only intermittently. The instrument itself is not deterministic, and the ripe verdicts rest on two or three runs of the same input per sequence, so a dataset would move to unstable if a later run of one of its sequences failed. The region scores of the photometric ablation come from one training seed. The requirement sweeps vary one property on two sequences, both of which are UAV row passes over one estate, so the thresholds they give are established for that geometry and transfer to others only through the spacing. Novel-view metrics on the every-eighth split overstate quality by the interpolation share that the block split exposes, and in the ablations, each condition is scored against its own processed frames, so that those comparisons measure consistency rather than fidelity, which is why registration leads every ablation table. Each mitigation was tested on the sequences of one dataset, two Botrytis flights, two GrapeMOTS passes and three BLT front passes, so the ablation results are existence proofs within that dataset rather than generalizations across datasets. The region metric covers GrapeMOTS only, since it is the one dataset with per-frame fruit masks. Lens artifacts were annotated by inspection by one person, without a second annotator to measure agreement. Scale anchors validate the shape and scale of a camera path against a reference whose own error is included in the residual, and for five datasets, no anchor exists at all. The study also ends at reconstruction, so it does not train a reinforcement-learning agent, run a simulation with physical interaction, or measure how any robotic task performs on the reconstructed scenes, which the conclusions return to as the next step. Finally, the measured properties are those of the sampled frames at a width of at most 1600 pixels under the fixed configuration, and a dataset’s readiness in Table 7 is readiness under that configuration, not a property of the data independent of the instrument. The schema does not measure fruit visibility, although picking depends on it. Where masks exist, it can be measured as the share of pixels inside grape clusters, which on the GrapeMOTS row passes is 0.7 to 1.7% of a held-out view, and elsewhere, it would have to be annotated. BTG’s bunch masks on 681 frames would allow the same region scores but were not computed.

## 8. Conclusions

This study measured whether the publicly available vineyard datasets can be reused for scene reconstruction. Of eighteen datasets found, ten were characterized and six contain overlapping views, and under one fixed instrument, three of the ten are ripe (BTG, GrapeMOTS and GrapeSLAM), whose sequences registered in every run made and reconstructed without treatment. Four are ripening and usable at a stated cost (Botrytis after band alignment, BLT across repeated runs, and EscaYard and VineLiDAR only as geometric priors). The remaining three are unripe, because single photographs cannot be registered whatever their resolution, although the video frames that WGISD ships beside its photographs reconstruct as well as the ripe datasets. Across the record, registration followed the capture rather than the dataset; novel-view quality varied as much within a dataset as between datasets; and frame spacing was the one capture property that limited reconstruction. Each mitigation holds within a stated boundary, since band alignment is required whenever the bands of a multi-lens sensor are composed into a color image and is unnecessary when a single band suffices for the reconstruction. Dehazing raises the PSNR of the fruit regions of a backlit pass by 0.7 to 1.5 dB on a single seed, a gain this study cannot separate from noise. Dehazing does not improve their SSIM or LPIPS and leaves both the frame as a whole and evenly lit passes unchanged. Masking the chassis of a ground robot does not consistently improve registration, and removing a standing tail left by a cutting fault reduces it.

The record should grow, and the online characterization is built so that a new dataset enters it under the same fixed configuration, which turns the gaps of Table 8 into a list of captures worth recording. The reconstructions should also be used, and the application that motivated this work, photorealistic environments in which agricultural robots are trained and evaluated, needs what the record mostly lacks: a metric scale, both canopy sides with a trajectory, and per-frame semantic masks. Integrating the ripe reconstructions into a simulation framework and measuring what a robotic task recovers from them is the experiment that these measurements make possible.

## Figures and Tables

**Table 1:** Datasets identified by the structured search. License is the license declared by the deposit, and not stated means it declares none. Public access is yes for an open download, after registration when the host asks only for an account or a form, and restricted when access can be refused. Docs is whether the acquisition is described in a data paper or a README. Size is the download size with the number of images, videos or point clouds. Characterized is whether the dataset was put through the measurement pipeline of this section. The datasets not characterized contain only single images without the overlap that reconstruction needs, except SynthRGB, *^b^* which is synthetic imagery. *^a^* The deposit states CC-BY-NC-SA-4.0 and the README CC-BY-NC-4.0.

Dataset	License	Public Access	Docs	Size	Characterized
BLT [20,51]	CC-BY-4.0	after registration	yes	9.1 GB subset (600 GB raw, multimodal)	yes
Botrytis [17]	CC-BY-4.0	yes	yes	31.1 GB (16,504 images)	yes
BTG [16,52]	CC-BY-4.0	yes	yes	21.7 GB (40 videos)	yes
EscaYard [13,53]	CC-BY-4.0	yes	yes	8.6 GB (multimodal)	yes
GrapeCS-ML [11,54]	not stated	yes	yes	631 MB (2327 files)	yes
GrapeMOTS [18,55]	CC-BY-4.0	yes	yes	87 GB (11 videos)	yes
GrapeSet [12,56]	CC-BY-4.0	yes	yes	878.4 MB (3910 files)	yes
GrapeSLAM [44,57]	CC-BY-4.0	yes	yes	40.4 GB (24 videos)	yes
VineLiDAR [19,58]	CC-BY-4.0	yes	yes	2.8 GB (10 point clouds)	yes
WGISD [15]	CC-BY-NC-SA-4.0 *^a^*	yes	yes	1.3 GB (300 images)	yes
GBVT [50]	CC-BY-4.0	yes	no	3.6 GB (1300 images)	no
GISV [47]	MIT	yes	no	970 images	no
GLD [45]	CC0: Public Domain	yes	limited	4195 images	no
GLHD [14]	CC-BY-4.0	yes	limited	2.8 GB (18,000 images)	no
GrapeNet [10]	CC-BY-4.0	yes	yes	4.4 GB (25,425 images)	no
LDD [46]	CC-BY-NC-SA-4.0	restricted	yes	29.6 GB (1092 images)	no
SynthRGB [49]	not stated	yes	no	2 GB (1000 images)	no *^b^*
UAV-VLIS [48]	CC-BY-SA-4.0	yes	no	2256 images	no

**Table 2:** Properties recorded for every characterized dataset. The Question column groups the properties by what they describe, the scene, the observation, the recording and the yield, which is what reconstruction recovers from the data. Each measure or unit begins with its data type, one of category, count, measurement, date or text. Documented values come from the data paper, repository record or README; inferred values follow from documented ones; measured values are obtained from the data with the procedure described in the text; and annotated values come from inspection of the frames.

Question	Property	Source	Measure or unit
Scene	Site	documented	categorical, estate or block
	Shared with other datasets	documented	text, names of the datasets
	Acquisition dates	documented	date
	Temporal span	documented	measurement, days to months
	Campaigns or revisits	documented	count
	Phenological stage	documented, inferred	categorical, code on the Biologische Bundesanstalt, Bundessortenamt und CHemische Industrie (BBCH) scale, or stage name
	Spatial extent	documented	measurement, ha, rows or plants
	Disease or stress state	documented	categorical, named condition
Observation	Platform	documented	categorical, UAV, ground robot, handheld, tripod
	Camera height or flight altitude	documented, measured	measurement, m above ground, from the Exchangeable Image File Format (EXIF) metadata where available
	View direction	documented, inferred	categorical, nadir, oblique, horizontal
	Canopy sides covered	documented, inferred	categorical, one or both
	Sequence continuity	documented	categorical, video, interval stills, isolated stills
	Number of sequences	documented	count
	Frame rate	documented, measured	measurement, Hz
	Illumination and weather	documented	text, as stated
	Frames per sequence	measured	count
Recording	Modality	documented	categorical, RGB, multispectral, RGB and depth (RGB-D), LiDAR
	Image resolution	documented, measured	measurement, pixels
	Intrinsics and distortion model	documented	categorical, provided, EXIF only, none
	Radiometric calibration	documented	categorical, panel images, applied, none
	Metric anchoring	documented	categorical, real-time kinematic (RTK) positioning, ground control points (GCPs), global navigation satellite system (GNSS) tags, none
	Trajectory or inertial measurement unit (IMU)	documented	categorical, provided or none
	Auxiliary 3D data	documented	categorical, LiDAR cloud, SfM cloud, none
	Annotations	documented	text, type and count
	License and access	documented	categorical, license and access route
Yield	Geometric recoverability	measured	measurement, share of frames registered
	Registration stability	measured	measurement, success rate over repeated runs
	Reconstruction connectivity	measured	count, disconnected models
	Feature persistence	measured	measurement, mean track length, frames
	Parallax adequacy	measured	measurement, median triangulation angle, degrees
	Viewpoint coverage	measured	measurement, solid angle of viewing directions, sr
	Frame degradation	measured	measurement, share of blurred frames and share of clipped pixels
	Static content	measured	measurement, share of pixels static over the sequence
	Documentation reliability	measured	text, agreement of documented values with the data
	Lens artifacts	annotated	categorical, glare, ghosting, fringing, none

**Table 3:** Novel-view quality of the reconstructions, one row per reconstructed sequence, grouped by dataset. Every input is untreated except the Botrytis flights, which are reconstructed from the aligned color composite of Section 5.1. Each sequence is scored on two held-out splits: the every-eighth split, which holds out every eighth frame, and the block split, which holds out contiguous blocks of four frames and is the harder test. Reg. is the registered frames over the frames given to the solver. Views is the number of held-out views on the every-eighth split then the block split. PSNR, SSIM and LPIPS are each averaged over the held-out views, and for PSNR, the spread across those views is also given in dB. nn Dist. measures how far the held-out views sit from the training views, as the mean distance from a held-out view to its nearest training view divided by the length of the camera path and given as a percentage on the every-eighth split then the block split. A small value means the held-out views have close training neighbors and are easy to synthesize, and a larger value a harder test. Sequences are named as in their deposits. GrapeMOTS row passes are its NoPathPlanning videos, and the orbits are its PathPlanning videos. Botrytis flights are named by camera tilt and visit, where visit 1 is the morning and visit 2 the afternoon nadir flight. Rows within a group share one flight, one plot or one block and are therefore not independent samples.

			Every-Eighth Split			Block Split			
Sequence	Reg.	Views	PSNR	SSIM	LPIPS	PSNR	SSIM	LPIPS	nn Dist., %
BTG, one flight on 28 June 2021									
row 4.1, part 2	45/45	6/4	22.9 ± 3.3	0.85	0.09	26.2 ± 1.0	0.92	0.05	2.0/0.7
row 4.2, part 3	29/29	4/4	20.0 ± 3.7	0.73	0.16	19.1 ± 0.7	0.64	0.21	3.4/4.8
row 4.3, part 1	49/49	7/4	23.3 ± 3.7	0.86	0.08	23.3 ± 0.3	0.86	0.09	2.1/2.7
row 4.4, part 3	26/26	4/4	15.7 ± 3.3	0.47	0.30	17.6 ± 0.7	0.65	0.18	4.0/1.3
row 6.1, part 1	41/41	6/4	22.6 ± 2.7	0.84	0.10	21.4 ± 0.7	0.78	0.13	2.5/3.8
row 6.2, part 2	24/24	3/4	21.8 ± 3.5	0.85	0.10	22.6 ± 0.6	0.84	0.10	4.5/6.5
row 6.3, single part	24/24	3/4	17.2 ± 3.0	0.61	0.23	15.3 ± 0.8	0.44	0.31	4.1/6.5
row 7.1, part 3	47/47	6/4	19.5 ± 3.9	0.69	0.20	14.1 ± 1.1	0.51	0.34	1.9/3.5
row 7.2, part 3	66/66	9/8	24.1 ± 3.9	0.85	0.09	23.2 ± 3.1	0.81	0.12	1.5/2.5
row 7.3, part 2	45/45	6/4	20.6 ± 1.1	0.78	0.12	19.3 ± 0.6	0.73	0.15	2.1/3.2
row 7.4, part 2	46/46	6/4	15.6 ± 2.2	0.45	0.33	11.6 ± 1.1	0.27	0.51	2.2/3.2
row 8, part 1	60/60	8/8	23.2 ± 1.7	0.86	0.10	22.3 ± 1.0	0.83	0.12	1.5/2.6
GrapeMOTS, harvest 2023									
row pass 1	60/60	8/8	21.0 ± 1.8	0.69	0.18	16.4 ± 1.5	0.49	0.32	1.6/2.7
row pass 2	60/60	8/8	22.6 ± 2.0	0.82	0.12	20.9 ± 0.9	0.78	0.15	1.7/2.4
row pass 3	60/60	8/8	21.3 ± 2.3	0.79	0.13	20.4 ± 1.6	0.75	0.15	1.6/2.3
orbit 1	54/54	7/8	23.1 ± 1.8	0.70	0.22	23.1 ± 0.9	0.71	0.25	1.4/0.8
orbit 2	28/28	4/4	22.5 ± 0.4	0.70	0.20	18.7 ± 0.8	0.48	0.36	1.5/10.2
orbit 8	50/50	7/4	25.1 ± 1.7	0.77	0.22	20.9 ± 1.0	0.68	0.30	1.3/7.9
GrapeSLAM									
Side_Short_Light	52/52	7/4	20.2 ± 1.5	0.70	0.17	16.8 ± 0.4	0.48	0.30	1.8/3.5
Botrytis, four flights on 16 September 2021									
0° tilt, visit 1	188/200	24/23	21.5 ± 6.0	0.65	0.29	22.2 ± 6.8	0.66	0.27	0.1/0.1
0° tilt, visit 2	185/200	25/24	14.7 ± 3.7	0.45	0.51	17.2 ± 4.5	0.58	0.39	0.4/0.4
30° tilt, visit 1	172/199	22/19	15.5 ± 2.4	0.44	0.51	16.7 ± 3.5	0.52	0.44	0.2/0.2
45° tilt, visit 1	200/200	25/24	23.5 ± 4.5	0.78	0.21	23.1 ± 3.0	0.76	0.21	0.2/0.3
BLT									
Greece, 20 April 2022, side camera	141/141	18/16	20.8 ± 4.9	0.68	0.45	22.2 ± 0.8	0.68	0.40	0.7/1.1
Greece, 8 June 2022, side camera	90/136	11/12	10.3 ± 2.6	0.38	0.83	7.7 ± 2.3	0.27	0.83	1.0/1.6
UK, 26 July 2023, side camera	52/52	7/4	15.7 ± 3.0	0.51	0.55	14.7 ± 1.4	0.47	0.51	1.8/2.0
WGISD									
demonstration video	200/200	25/24	22.3 ± 1.8	0.68	0.24	20.4 ± 0.7	0.56	0.29	0.5/0.8

**Table 4:** Ablation of the multispectral band composition on two Botrytis flights, 200 frames each, grouped into baselines, the full method and its variants. The full method aligns each band to the green band with one median homography, applies a joint percentile stretch across the three bands and a gray-world white balance, and crops a 30 px border. Each variant changes one component of it, where a minus sign marks a removed component and the two unsigned variants change how a component is handled. The false-color composite of the NIR, red-edge and red bands is a baseline evaluated only on the 45° flight, as an alternative to the green band rather than as a step in the alignment pipeline. Registered is the number of frames in the largest model, with the number of models where it exceeds one, and PSNR is the mean over three training seeds on the held-out views of the every-eighth split.Each condition is scored against its own composite, so PSNR compares conditions on how consistently they render what they were given rather than on color fidelity, and the registered column is the comparison that does not depend on the composite.

	45° Flight		Nadir Morning Flight	
Condition	Registered	PSNR	Registered	PSNR
blue, green and red bands stacked as recorded	109 (2 models)	18.0	179	16.6
green band alone	200	25.9	190	23.6
near-infrared (NIR), red-edge and red false-color composite	200	21.2	–	–
**Full method**	200	23.4	188	21.1
−alignment	109 (2 models)	18.2	141 (2 models)	15.3
−radiometry (stretch and white balance), alignment only	200	19.7	189 (2 models)	16.4
−border crop	200	20.2	189	17.1
alignment estimated per frame, not one median homography	200	18.9	181	16.4
joint stretch replaced by independent per-band stretch	200	22.2	187	19.6

**Table 5:** Ablation of the photometric treatment on the backlit GrapeMOTS row pass and on a row pass flown under even light as control, grouped into the pass as recorded, the full method and its variants. The full method applies dark-channel dehazing with guided-filter transmission and atmospheric light taken as the mean of the brightest 0.1% of pixels. A plus sign marks a component added to it and a minus sign a component removed, the single-brightest-pixel variant changes how the atmospheric light is estimated, and the last row applies the lens-ghost mask without dehazing. Every condition registers all 60 frames, so the columns give novel-view quality only: PSNR over the full frame as the mean of three seeds, and for the backlit pass also PSNR, SSIM and LPIPS inside the dataset’s grape-cluster masks and over the remaining canopy for the first seed. Each condition is scored against its own treated frames rather than against the recorded ones.

	Backlit Row Pass 1							Row Pass 2
	Frame	Clusters			Canopy			Frame
Condition	PSNR	PSNR	SSIM	LPIPS	PSNR	SSIM	LPIPS	PSNR
as recorded	20.8	23.8	0.77	0.20	20.9	0.68	0.19	22.6
**full method**	20.2	24.5	0.77	0.20	20.1	0.67	0.18	22.6
+lens-ghost mask	20.2	25.2	0.78	0.19	20.0	0.67	0.18	22.5
−guided filter	19.9	24.2	0.73	0.21	19.9	0.66	0.19	22.4
atmospheric light from the single brightest pixel, not the brightest 0.1%	20.5	25.3	0.78	0.19	20.4	0.68	0.18	22.6
lens-ghost mask only, no dehazing	20.8	22.7	0.74	0.24	20.2	0.66	0.22	22.6

**Table 6:** The BLT front camera across masking approaches and frame cuts. The table has two parts. The first, on the UK pass, compares masking approaches: no mask, a fixed-region mask and a color-threshold mask (both automatic). The fixed-region mask registers in none of five runs, and the color-threshold mask was run only once, so neither is shown to help, and the hand-drawn manual mask (Figure 10) is adopted as the chassis mask used throughout the second part. The second part, on the Greek passes, applies no mask or that chassis mask across cuts of the same recording. Cut is which selection of frames was reconstructed; the pass as originally sampled, which for 13 July and 15 September ends in a standing tail where the robot had stopped while recording (Figure A3), the same pass with that tail trimmed, or the pass re-cut on the time stamps of the images. Frames is the number of sampled frames. Runs registered is the number of repeated registration runs whose largest model holds at least half the frames, out of five where the condition was repeated and out of one otherwise. PSNR is the mean in dB over three training seeds of one registered run, and a dash means no trained result, either because no run passed the registration gate or because the pass was put through registration only. For 13 July and 15 September, PSNR is measured on the held-out views of the every-eighth split that the trimmed passes keep (17 and 12 views), and the trimmed rows are one training of the run that registered. For the other passes, it covers all held-out views of the every-eighth split.

Front-Camera Pass	Cut	Condition	Frames	Runs Registered	PSNR
Choosing a mask					
26 July 2023, UK	as recorded	none	53	2 of 5	—
		fixed-region mask, 28.8%	53	0 of 5	—
		color-threshold mask, 18.8%	53	1 of 1	12.6
Isolating the cut					
23 March 2022, Greece	as recorded	none	137	1 of 1	21.9
20 April 2022, Greece	as recorded	none	142	1 of 1	18.5
8 June 2022, Greece	as recorded	none	137	1 of 1	16.3
13 July 2022, Greece	original, standing tail	none	148	1 of 5	—
		chassis mask (hand-drawn, 12.5%)	148	4 of 5	15.0
	original, tail trimmed	none	131	1 of 5	14.6
		chassis mask	131	0 of 5	—
	corrected	none	133	0 of 5	—
		chassis mask	133	0 of 5	—
15 September 2022, Greece	original, standing tail	none	116	2 of 5	16.1
		chassis mask	116	2 of 5	16.7
	original, tail trimmed	none	95	0 of 5	—
		chassis mask	95	1 of 5	16.6
	corrected	none	137	1 of 5	—
		chassis mask	137	2 of 5	—

**Table 7:** Readiness of the characterized datasets for reconstruction under the fixed configuration, together with the measured values on which each verdict rests. Readiness names the class defined in Section 6.1, with ripe, ripening or unripe in brackets, each marked with its color in Figure 1. Registered is the share of frames that the largest model holds, and stability the number of registered runs out of the runs made on the same input, from Figure 11 where a dataset has an input there and otherwise from its characterization and reconstruction runs. PSNR is the range on the every-eighth split over the n reconstructed sequences of the dataset. Scale anchor names the external reference shipped with the dataset and, where a reconstructed camera path could be aligned to it, the alignment error, which includes the error of the reference itself. The limit column names the measured property that sets the class or, for a ripe dataset, the caveat that applies to it. The evidence column gives the tables, figures, and sections from which the row is taken. Dashes mark values that do not apply to a dataset without registered imagery.

Dataset	Readiness	Registered, %	Stability	PSNR, dB (n)	Scale Anchor	Limit or Caveat	Evidence
BTG	ready (■ ripe), per part	100 per part, 40 per clip	3 of 3	15.6 to 24.1 (12)	none deposited	parts of a clip do not join	Table A4 and Table 3, Figure 11
GrapeMOTS	ready (■ ripe)	100	3 of 3	21.0 to 25.1 (6)	none deposited	one pass backlit	Table A4, Table 3 and Table 5
GrapeSLAM	ready (■ ripe)	100	2 of 2	20.2 (1)	flight log, 5.6 cm	one of 24 videos characterized	Table A4 and Table 3, Section 4.4
Botrytis	ready with treatment (■ ripening)	89 to 100, green band	1 of 1	14.7 to 23.5 (4), aligned composite	EXIF GPS, about 1 m	bands need alignment for RGB	Table A4 and Table 4
BLT	unstable (■ ripening)	1 to 100, by session	0–2 of 5	10.3 to 20.8 (3)	RTK, 7 to 10 cm	front camera registers in 0 to 2 of 5 runs	Table A4 and Table 6, Figure 11
EscaYard	prior only (■ ripening)	0.8	–	–	EXIF GPS, trunk survey	products shipped, not frames	Table A4
VineLiDAR	prior only (■ ripening)	no imagery	–	–	coordinate system, 8 of 10 files	point clouds only	Table A4
WGISD	not ready (■ unripe), video frames ready	0.7 stills, 100 video	2 of 2	22.3 (1), video	none	the photographs are single views	Table A4 and Table 3
GrapeCS-ML	not ready (■ unripe)	3.2	–	–	none	single views	Table A4
GrapeSet	not ready (■ unripe)	5.0	–	–	none	rotated objects, fixed camera	Table A4

**Table 8:** Combinations of properties that no characterized dataset provides, each read from a pair of empty cells in Table A3 and Table A4, grouped into captures that do not exist and data that exist but were not deposited or documented. The first column is the missing combination, the second what a dataset with it would make possible, and the third what it would cost beyond the data that already exist. The table is a shortlist of the captures that would most extend the record.

Absent from the Record	What It Would Make Possible	Cost Beyond Existing Data
Missing captures, which need a new recording		
a UAV capture of one block in more than one season	reconstruction of canopy growth and fruit development on the same plants. At present only the BLT ground robot provides this.	repeating an existing flight plan three to four times a year
a ground-level capture outside Greece and the UK	side-view reconstruction of the trellis systems and cultivars that the UAV datasets show only from above	one robot or handheld session per site
surveyed GCPs discernible at the flight height	absolute georeferencing of a UAV reconstruction. The Botrytis targets cannot be identified at 20 m.	larger targets, or a lower flight over the same points
a capture of the dormant season	reconstruction of the exposed trellis and cane structure. The first BLT sessions touch it, and no UAV dataset does.	one flight in winter or early spring
a video or interval capture with the single-view annotations of the stills sets	projection of cultivar, disease and berry annotations into a 3D model	recording the walk between the photographs that the handheld datasets already took
Missing deposits or documentation of data that already exist		
a UAV row pass covering both canopy sides with a deposited trajectory	a metrically anchored model of a whole row. At present GrapeMOTS covers both sides without a trajectory and GrapeSLAM ships a trajectory for one side only.	exporting the flight log that an RTK platform already records
LiDAR and overlapping imagery of one canopy state in one coordinate system	a geometric reference for every image-based reconstruction. The same-day Botrytis and VineLiDAR captures would give one if the LiDAR files carried a coordinate system.	a coordinate system in two existing files
measured view overlap or spacing between consecutive frames	selection of a dataset for reconstruction from its documentation alone, without the measurement of Section 3	one line in the data paper

**Table 9:** Recommendations for dataset creators and users, each resting on a measurement of this study and grouped by audience: creators who record new datasets, users who reconstruct existing ones, and both. For each recommendation, the measured basis gives the result it rests on, and the last column points to where that result is reported. The thresholds hold for standard SfM followed by 3DGS and for the capture geometries of the characterized datasets and are not claimed beyond them.

Recommendation	Measured Basis	Reported in
For dataset creators		
Record consecutive frames no more than about 3% of the path apart, which at the flight speeds of the record means at least two frames per second.	At 0.5 frames per second, the backlit GrapeMOTS pass registers 2 of 15 frames and fails in all five runs, whereas at two frames per second both test passes register in full.	Section 4.5, Table A2
Cover both sides of each row.	A pass along one side of a row reconstructs a façade rather than a volume, and no characterized UAV row pass covers both sides with a trajectory.	Section 3.4, Table 8
Deposit the camera trajectory or flight log together with the imagery.	The GrapeSLAM flight log aligns to its reconstruction within 5.6 cm, whereas BTG and GrapeMOTS state an RTK platform but deposit no trajectory.	Section 4.4
On a ground robot, record with a sideways camera and cut each pass on image timestamps.	The BLT side camera registers in four runs of five against zero to two for the front camera, and two BLT passes selected on recording time instead of image time stamps end with 17 and 21 standing frames.	Section 5.3, Figure 11
For both creators and users		
Record at any width from 640 pixels upward, and downsample native 4K frames to 1600 pixels or less before reconstruction.	Training at 640 pixels costs at most 1.1 dB against 960 or 1600 pixels, and native 3840 or 4096 pixels lose 0.3 to 0.9 dB.	Section 4.5
Align the bands of a multi-lens sensor with one homography per band before composing a color image, or deposit the band calibration.	Alignment raises the registered frames of the 45° Botrytis flight from 109 to 200, and a homography per frame fails on 14 to 37% of the frames.	Section 5.1, Table 4
Avoid passes against a low sun, because dehazing does not measurably repair a backlit pass.	Dehazing raises the cluster PSNR of the backlit pass by 0.7 to 1.5 dB on one seed, but not its SSIM or LPIPS, and does not improve the frame as a whole or the evenly lit pass.	Section 5.2, Table 5
State the acquisition date, coordinate system, and license of every file, and check documented values against the data.	Eight of the ten characterized datasets contain at least one documented value that disagrees with the data, one states no acquisition date, and one states no license.	Section 3.4, Section 3.5 and Section 3.6, Table A4
For dataset users		
Register every capture at least twice, and an unstable capture at least five times, before reporting a result.	Two runs on identical input need not register alike, and the BLT front passes register in zero to two runs of five.	Section 4.2, Figure 11
Evaluate novel views on a block split in addition to the every-eighth split.	The block split lowers the score by more than 1 dB on 13 of the 27 reconstructions, by up to 5.4 dB, and exposes artifacts that the every-eighth split hides.	Section 4.3, Figure 6

**Figure A4:**
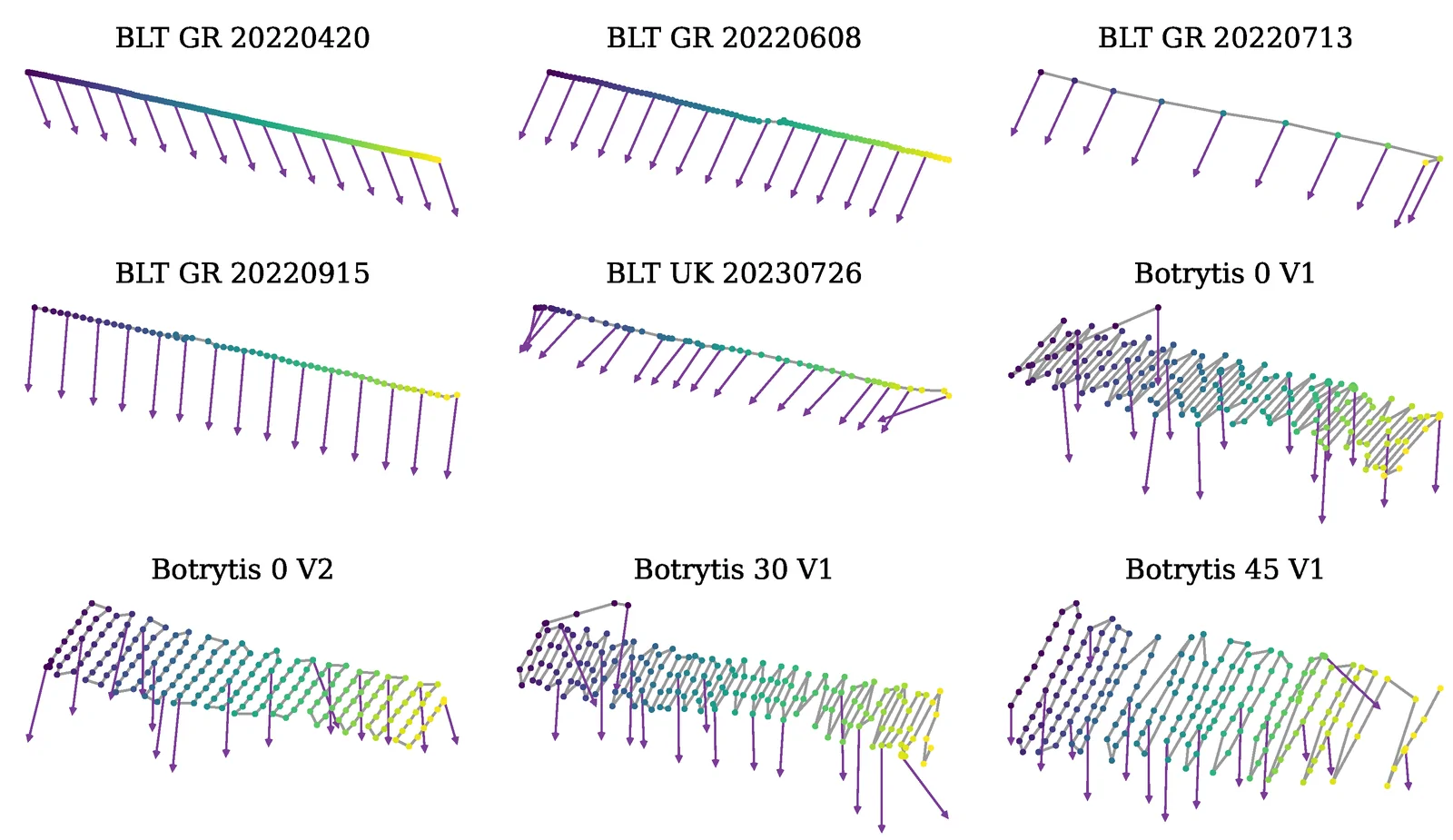

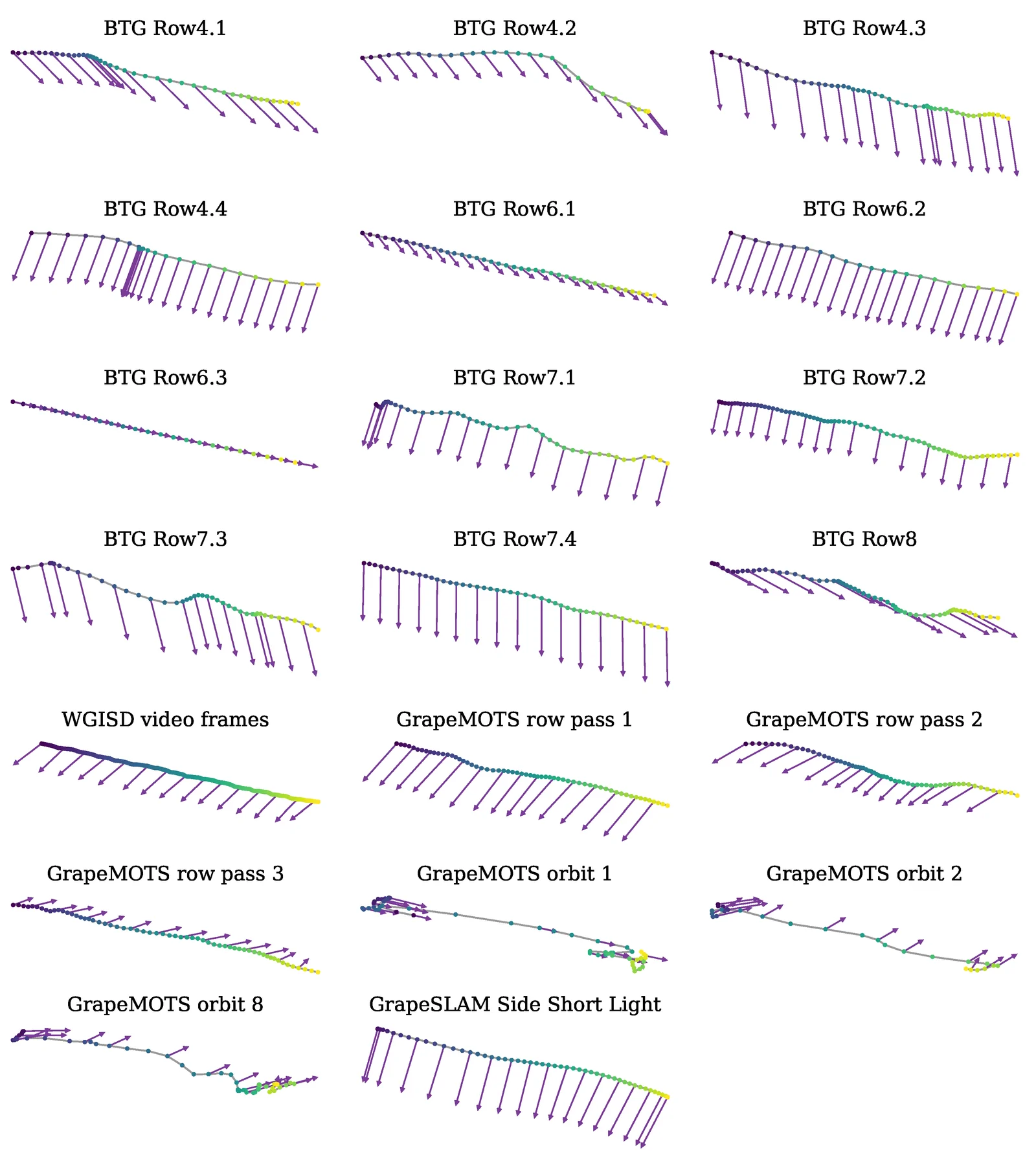
The complete atlas of registered camera paths, with one panel per characterized sequence, for all 29 sequences that reach a registered model. Each panel follows the conventions of Figure 3: every point is a camera, colored from the first frame (purple) to the last (yellow), with arrows on every few cameras showing the viewing direction, and each path is drawn in the principal axes of its own reconstruction and scaled to a common length, so panels compare in shape but not in size. Together, they show the range of capture geometries across the record, from the near-parallel views of UAV row passes, through the converging views of the BLT robot, to the perpendicular side-looking paths of the GrapeMOTS orbits. Sequences that register only in part, such as BLT Greece 13 July 2022, show only their registered cameras.

## Data Availability

No new datasets were created in this study. Nine of the ten characterized datasets are publicly available from their original repositories, namely WGISD [15], GrapeCS-ML [11], GrapeSet [12], BTG [16], GrapeMOTS [18], Botrytis [17], GrapeSLAM [44], EscaYard [13], and VineLiDAR [19], and the BLT dataset [20] is available after registration. The characterization is published in the online catalog at https://dragons-project.github.io/grapes-ready-for-picking/ (accessed on 31 August 2026), and the code, the reconstruction configurations and the catalog source are in the repository at https://github.com/DRAGONS-Project/grapes-ready-for-picking (accessed on 31 August 2026). The frame lists and registration records are available from the corresponding author on request.

## Appendix A. The Protocol as Run

This appendix records the configuration of every experiment in the paper as it ran, so that the rows of its tables can be reproduced from the public datasets and the code repository without consulting either for a parameter. Table A1 lists the configuration of every reconstruction row.

**Sampling:** Each sequence of *N* source frames is sampled at frames 0,k,2k,…, where the step *k* is the larger of ⌊N/200⌋ and half the frame rate rounded to an integer (and at least 1), and the first 200 sampled frames are kept, so a sequence gives at most 200 frames at no more than two per second. The cap shortens only the four Botrytis flights and the WGISD video, which therefore cover the first 200 sampled frames of their capture. Frames are resized to a width of 1600 pixels, never upscaled, and written as PNG. The resulting frame list of each sequence is fixed once written and reused by every later experiment on that sequence. BLT sequences use one corridor pass selected from the robot’s odometry as the longest run with speed above 0.4 m/s and heading change below 15°, and on 13 July and 15 September 2022, where the odometry was written to the recording 7 and 12 s after the images, the same row is re-cut on the time stamps of the images, and front-camera sequences reuse the side-camera window verbatim. Frames with a mean luminance of less than 8 are excluded before sampling. BTG sequences are parts, one source file each, and the quality rows use the largest part of every clip. Botrytis composed sequences use exactly the captures of the green-band lists.

**SfM:** We use pycolmap 4.0.4 on the central processing unit (CPU) with the random seed fixed to 0, as well as scale-invariant feature transform (SIFT) features with a maximum image size of 1600 pixels and at most 8192 features per image, with the OPENCV camera model shared by all frames of a sequence, exhaustive matching, and the incremental mapper at its defaults. Optional masks follow COLMAP’s convention of zero marking pixels without features. A sequence passes the gate for training when its largest model holds at least half of its frames, and every run keeps its registration row regardless of the gate. The quality rows were gated on the characterization run and trained on the frames that their own run registered. After the gate, the best model and its frames are undistorted to a pinhole camera with pycolmap’s defaults, and loss masks are undistorted with the same model.

**Trainer:** We use gsplat 1.4.0 for 30,000 iterations, with one view per iteration with the training views shuffled each epoch and seed 0 unless stated. Gaussians are initialized at the sparse points with an isotropic size equal to the root mean square distance to the three nearest points, opacity 0.5, RGB color without spherical harmonics and identity rotation. Densification follows gsplat’s default strategy: growth thresholds 0.0002 on the screen-space gradient, 0.01 and 0.05 on the 3D and 2D scales, pruning below opacity 0.005 and above scale 0.1, refinement every 100 iterations from iteration 500 to 15,000, opacity reset every 3000, with no cap on the number of Gaussians. Adam optimizers with epsilon $10^{-15}$ use learning rates of $1.6\times 10^{-4}$ times the scene scale for positions, decaying exponentially to one percent over the run, $5\times 10^{-3}$ for scales, $10^{-3}$ for rotations, $5\times 10^{-2}$ for opacities and $2.5\times 10^{-3}$ for colors, where the scene scale is the 95th percentile of the distance of the sparse points from their centroid. The loss is $0.8\;L_{1}+0.2\;\left(1-\mathrm{SSIM}\right)$. Images train at the undistorted resolution. Where a loss mask is given, excluded pixels take the target’s value before the loss and carry no gradient.

**Evaluation:** Held-out views are fixed by image name per sequence and reused across all conditions. The every-eighth split holds out every 8th name from index 0, and the block split holds out blocks of 4 consecutive names starting at index 16 and every 32 thereafter, dropping a block that touches the sequence end, with sequences under 36 frames holding out their middle 4. Held-out names that did not register are skipped and counted. PSNR and SSIM come from scikit-image with a data range of one, and LPIPS from lpips 0.1.4 with the AlexNet backbone. For every held-out view, the distance from its camera center to the nearest training camera as a fraction of the camera path length and the angle between their optical axes are recorded. Masked scoring computes PSNR from the masked mean squared error and SSIM and LPIPS as their per-pixel maps averaged over the mask, with SSIM over the mask minus the three-pixel image border so that a full mask reproduces the unmasked value. The grape-region metric uses the GrapeMOTS instance maps, grape class only, resized and undistorted with the row’s camera.

**Preprocessing conditions:** Dehazing uses the dark-channel prior with a patch radius of 7, ω=0.90 and $t_{0}=0.15$, with the veil color as the mean of the brightest 0.1% of dark-channel candidates or the single brightest candidate in the ablation variant and transmission refined by a guided filter of radius 40 and $\epsilon =10^{-3}$. Lens-ghost masks mark pixels whose excess green exceeds a baseline over a 45-pixel radius and whose value channel texture over a 5-pixel radius is low, with a value floor of 0.45, keeping blobs of at least 0.2% of the frame dilated by 9 pixels. Botrytis composition aligns blue and red onto green by one homography per band, the median over 25 captures of Oriented FAST and Rotated BRIEF (ORB) correspondences filtered by random sample consensus (RANSAC) with at least 80 inliers each, or by a homography per frame with identity on failure, stretches bands jointly or independently to the 2nd and 98th percentiles, applies a gray-world white balance, and crops a 30-pixel border. The full method is median alignment, joint stretch, white balance and crop.

**Scale anchors.** Registered camera centers are aligned to the reference positions by a similarity transform estimated with Umeyama’s method, and the residual is the root mean square camera-position error after alignment. BLT references are the RTK fixes, all with fix status 2, interpolated to each frame’s header time in a local east-north-up frame. Botrytis references are the EXIF GPS positions of the captures. The GrapeSLAM reference is the shipped flight log at 10 Hz, with frame time taken as the frame index over 30 and a time offset searched from −4 to +2 s, and this one alignment was computed interactively, so its code is not in the repository.

**Sweeps and repeats:** A sweep varies one property with everything else at the protocol, one seed, scored on the every-eighth split of its own frame list and additionally with renders and references resized to 640 pixels. Width levels above 1600 pixels change training and scoring only, since feature extraction stays capped. The repeat study runs the fixed configuration on identical frames and masks with the random seed and the hash seed set to the run index and four threads. Nothing else is changed, and a run counts as registered when its largest model holds at least half of the frames.

**Hardware, software and noise floor:** Graphics processing unit (GPU) jobs ran on one NVIDIA H100 with two to four CPU cores and 32 to 48 GB of memory on the PLGrid Lem system, and CPU stages on one to eight cores. The software used was Python 3.11, PyTorch 2.12 with CUDA 13.0, gsplat 1.4.0, pycolmap 4.0.4, lpips 0.1.4, OpenCV 4.13 and scikit-image. A 60-frame sequence at 1600 pixels takes about 96 s of SfM on 8 cores, 10 s of undistortion, 660 to 720 s of training to 7 to 14 million Gaussians within 7 GB of GPU memory, and 8 s of evaluation before LPIPS. Repeating a training with the same seed changes the mean PSNR by 0.02 dB. Another seed changes the mean by 0.3 dB in the median over the 37 ablation conditions trained with three seeds, by up to 0.9 dB on the UAV sequences and by up to 1.5 dB on the BLT front passes, and a single held-out view by up to 1.5 dB, so that differences below about 1 dB between single runs are not evidence and ablation conditions were run with three seeds. Training on distorted frames instead of undistorted ones cost 2.5 dB in the check that preceded these experiments. The code is at https://github.com/DRAGONS-Project/grapes-ready-for-picking (accessed on 31 August 2026), and the frame lists, registration records, and per-view metrics of every row are available from the corresponding author on request.

**Table A1:** Configuration of every reconstruction row of Table 3. Frames available refers to the length of the source sequence after the pass window was cut; step refers to the sampling stride in source frames; spacing refers to the resulting interval between consecutive sampled frames, with a dash where the frames carry no frame rate; given refers to the frames handed to the solver; registered refers to those in the largest model and trained on; reproj. refers to the mean reprojection error of that model in pixels; points refers to its number of sparse points in thousands; held out refers to the test views of the every-eighth and the block split; Gaussians refers to the final count of the every-eighth model in millions; and training refers to its wall time on one H100 for 30,000 iterations. A BTG clip such as row 4.1 concatenates several separate recordings, and since each registers as its own model, the largest of them, its part, is the unit reconstructed here.

Sequence	Frames Available	Step	Spacing, s	Given	Regis-Tered	Reproj., px	Points, k	Held Out	Gaussians, M	Training, s
BTG row 4.1 part 2	1348	30	0.50	45	45	0.42	36.3	6/4	7.2	692
BTG row 4.2 part 3	866	30	0.50	29	29	0.32	24.3	4/4	9.4	760
BTG row 4.3 part 1	1467	30	0.50	49	49	0.44	38.7	7/4	6.2	672
BTG row 4.4 part 3	776	30	0.50	26	26	0.49	9.7	4/4	6.7	657
BTG row 6.1 part 1	1211	30	0.50	41	41	0.41	39.0	6/4	7.7	749
BTG row 6.2 part 2	702	30	0.50	24	24	0.35	21.2	3/4	4.1	596
BTG row 6.3	717	30	0.50	24	24	0.35	12.8	3/4	10.6	766
BTG row 7.1 part 3	1390	30	0.50	47	47	0.36	37.7	6/4	12.6	736
BTG row 7.2 part 3	1961	30	0.50	66	66	0.36	70.6	9/8	9.0	700
BTG row 7.3 part 2	1347	30	0.50	45	45	0.52	35.1	6/4	6.6	661
BTG row 7.4 part 2	1377	30	0.50	46	46	0.34	15.0	6/4	20.4	845
BTG row 8 part 1	1781	30	0.50	60	60	0.50	44.6	8/8	5.6	627
GrapeMOTS row pass 1	899	15	0.50	60	60	0.39	16.6	8/8	8.9	661
GrapeMOTS row pass 2	900	15	0.50	60	60	0.46	41.7	8/8	8.9	696
GrapeMOTS row pass 3	900	15	0.50	60	60	0.45	36.6	8/8	9.0	751
GrapeMOTS orbit 1	801	15	0.50	54	54	0.79	31.9	7/8	3.5	639
GrapeMOTS orbit 2	418	15	0.50	28	28	0.68	17.6	4/4	3.0	623
GrapeMOTS orbit 8	750	15	0.50	50	50	0.55	25.8	7/4	3.2	607
GrapeSLAM Side_Short_Light	769	15	0.50	52	52	0.39	22.8	7/4	13.9	819
Botrytis 0° visit 1	–	–	–	200	188	0.31	125.3	24/23	26.5	770
Botrytis 0° visit 2	–	–	–	200	185	0.33	146.2	25/24	25.1	774
Botrytis 30° visit 1	–	–	–	199	172	0.29	86.6	22/19	20.4	695
Botrytis 45° visit 1	–	–	–	200	200	0.34	130.0	25/24	15.3	651
BLT Greece 20 Apr 2022	985	7	0.47	141	141	0.60	26.4	18/16	0.9	589
BLT Greece 8 Jun 2022	951	7	0.47	136	90	0.71	9.0	11/12	0.6	417
BLT UK 26 Jul 2023	104	2	0.59	52	52	0.77	8.5	7/4	1.2	472
WGISD video	500	2	–	200	200	0.86	95.7	25/24	8.5	710

**Table A2:** Level-by-level results of the requirement sweeps summarized in Section 4.5, measured on two UAV row passes. One property is varied at a time while the other two stay at the protocol value, and every result is scored at a common width of 640 pixels so that the levels of a sweep are comparable. Spacing is the median distance between consecutive registered cameras as a share of the camera path, the quantity the solver depends on and which frames per second only stands for at a given flight height and speed. Registered is the share of frames that the largest model holds, with the number of models given where it exceeds one, PSNR is in dB, and a dash marks a level at which no value could be measured. The line that opens each block states the range of the varied property across the characterized datasets. At the protocol level, the frame count is the full sequence, i.e., 60 frames for the GrapeMOTS pass and 66 for the BTG part, so the GrapeMOTS pass has no separate 60-frame level, and the BTG 60-frame level is a separate run on 60 of its 66 frames.

	GrapeMOTS Row Pass 1, Backlit			BTG Row 7.2, Part 3		
Level	Spacing, %	Registered, %	PSNR	Spacing, %	Registered, %	PSNR
Frames sampled per second. Record: 1 (Botrytis) to 60 (BTG)						
0.5	–	13	–	6.3	100	14.3
1	4.0	80 (2 models)	16.1	2.9	100	20.1
**2 (protocol)**	1.6	100	22.1	1.4	100	25.6
4	0.8	100	26.2	0.7	100	29.1
Training image width, px. Record: 640 (GrapeCS-ML) to 4096 (BTG)						
640	1.6	100	21.0	1.5	100	26.3
960	1.6	100	22.0	1.4	100	26.1
**1600 (protocol)**	1.6	100	22.1	1.4	100	25.6
native, 3840 or 4096	1.6	100	21.2	1.4	100	25.3
Frames in the sequence. Record: 40 (GrapeSet) to 1961 (BTG)						
15	3.9	100	21.0	2.1	100	25.2
30	2.7	100	22.0	1.9	100	28.0
60	–	–	–	1.5	100	26.8
**full sequence (protocol)**	1.6	100	22.1	1.4	100	25.6

## Appendix B. Additional Qualitative Results

This appendix shows additional held-out views and camera paths that support statements of the main text. Figure A1 shows a BTG part at the two extremes of the frame-spacing sweep of Section 4.5. Figure A2 shows two Greek BLT front-camera passes from March and June, between which the reconstruction quality declines with the growing canopy (Section 5.3). Figure A3 shows the motion of the robot along the two BLT front passes whose original cut ends in a standing tail, and Figure A4 shows the registered camera paths of every characterized sequence. In the figures of held-out views, the render is on the left, the ground-truth frame is on the right, and the score given is that of the view shown.

## Appendix C. Characterization Tables

This appendix lists the documented and measured properties of the ten characterized datasets under the schema of Table 2. Table A3 gives the scene and observation properties, and Table A4 gives the recording properties and the measured yield, which Section 3 summarizes in Figure 2. Both tables are also published as machine-readable files in the online catalog of Section 6.4.

**Table A3:** Scene and observation properties of the ten characterized datasets. Dashes mark properties that do not apply, and not stated marks values the documentation does not give. Measured values are medians over sequences with their range in brackets, and where a dataset has two groups (GrapeMOTS row passes and orbits, BLT Greek (GR) and UK sessions) both are given, separated by a slash.

Property	BTG	Botrytis	GrapeMOTS	GrapeSLAM	BLT	EscaYard	VineLiDAR	WGISD	GrapeCS-ML	GrapeSet
Site	Tomiño B9	Tomiño B9	Tomiño B9	Tomiño B9	Gerovassiliou GR/Riseholme UK	Tomiño B7, B9	Tomiño B7, B9	Guaspari, Brazil	3 Australian vineyards	indoor (USA)
Shared with	Botrytis, GrapeMOTS, GrapeSLAM, EscaYard, VineLiDAR	BTG, GrapeMOTS, GrapeSLAM, EscaYard, VineLiDAR	BTG, Botrytis, GrapeSLAM, EscaYard, VineLiDAR	BTG, Botrytis, GrapeMOTS, EscaYard, VineLiDAR	–	BTG, Botrytis, GrapeMOTS, GrapeSLAM, VineLiDAR	BTG, Botrytis, GrapeMOTS, GrapeSLAM, EscaYard	–	–	–
Dates	28 June 2021	16 September 2021	19–20 September2023	18–19 September 2023	March–September 2022/July–Aug 2023	14–15 July 2022	September 2021/July, September 2022	April 2017/April 2018	January–May 2017	not stated
Temporal span	1 day	1 day	2 days	2 days	7 months/3 weeks	2 days	3 dates	2 dates	4 months	–
Campaigns	1	1	1	1	10/5	1	3	2	7 rounds (Set 1)	1
Phenological stage	fruit set to veraison (BBCH 75–79)	harvest	harvest	harvest	dormant to full canopy/not stated	fruit set to veraison (BBCH 77–79)	vegetative growth (Jul), near harvest (Sep)	not stated	veraison to maturity	–
Spatial extent	4 rows	1.06 ha	3 rows/8 vines	24 videos	5 corridors	2 blocks, 42 plants	2 blocks	5 plots	3 vineyards	10 bunches
Disease state	early Botrytis	Botrytis per cluster	–	–	–	esca per plant	–	–	–	–
Platform	UAV	UAV	UAV	UAV	ground robot	UAV/smartphone	UAV	handheld	handheld	tripod
Height above ground	3 m	30 m stated/ 16–20 m by EXIF	3 m	1–3 m	ground	30 m/1–2 m	20–50 m	1–2 m	not stated	–
View direction	60° tilt, side	0/30/45° off nadir	side, frontal/orbits	front/side	forward/sideways	nadir/horizontal	nadir	horizontal	not stated	horizontal
Canopy sides	one	–	both	not stated	both	–	–	–	–	–
Sequence continuity	video	stills, 1 s	video	video	video (rosbag)	stills	point clouds	stills/video (repo)	stills	stills
Sequences	40 videos, 12 clips	4 flights	11 videos	24 videos	15 sessions, 13 with cameras	253 photos	10 clouds	300 photos/1 video	2327 files	3910 files
Frames per sequence	1348 per part [702–1961]	735 [526–1271]	900/750 [418–801]	769	951 GR/104 UK	–	–	500 (video)	–	–
Frame rate	59.94 fps	1 s interval	30 fps	30 fps	14.8 Hz GR/3.4 Hz UK	–	–	–	–	–
Illumination	clear	clear, afternoon flight	sunny/one backlit pass	varied	varied over season	midday, evening	afternoon	not stated	not stated	controlled

**Table A4:** Recording properties and measured yield of the ten characterized datasets. Registered share is the share of frames in the largest model under the fixed configuration; registration stability is the number of successful runs out of the runs made on the same input, from the repeated runs of Figure 11 (where a dataset has an input there) and otherwise from its characterization and reconstruction runs (or 1 of 1 where the input was registered once); and models is the number of disconnected reconstructions. Track length is given in frames; triangulation angle and viewpoint coverage are given as medians over sequences; and blurred frames, clipped pixels, and static content are given as shares. Lens artifacts are annotated by inspection, with unsure marking a tentative call, and documentation reliability lists the disagreements found between a dataset’s documentation and its data, with none marking the two datasets where no disagreement was found and the brackets giving their remaining documentation gaps.

Property	BTG	Botrytis	GrapeMOTS	GrapeSLAM	BLT	EscaYard	VineLiDAR	WGISD	GrapeCS-ML	GrapeSet
Modality	RGB	multispectral, 5 bands	RGB	RGB	2 RGB-D, LiDAR, IMU	RGB/multispectral products	LiDAR, RGB per point	RGB	RGB	RGB
Resolution	4096 × 2160	1280 × 960 per band	3840 × 2160 (two orbits 1920 × 1080)	3840 × 2160	1920 × 1080	4032 × 3024	9.6–71 M pts	2048 × 1365	640 × 480/ to 4032 × 3024	4032 × 3024
Intrinsics	–	EXIF	–	–	camera_info	EXIF	–	EXIF	EXIF	–
Radiometric calibration	–	panel captures	–	–	–	applied	–	–	color references	–
Metric anchoring	RTK stated, not deposited	7 GCPs/EXIF GPS	RTK stated, not deposited	flight log (GPS)	RTK-GNSS	EXIF GPS/trunk survey	CRS in 8 of 10 files	–	–	–
Trajectory or IMU	–	–	–	flight log	IMU, RTK	–	–	–	–	–
Auxiliary 3D	–	–	–	–	LiDAR clouds	SfM clouds	(primary)	–	–	–
Annotations	bunch masks, 681 fr.	cluster, trunk, GCP positions	tracking masks, 5958 fr.	–	path only	esca, cluster counts	–	boxes, masks, berries	chemistry/ masks (Set 5)	masks, counts, weights
License/access	CC BY/open	CC BY/open	CC BY/open	CC BY/open	CC BY/after registration	CC BY/open	CC BY/open	CC BY-NC(-SA)/ open	not stated/open	CC BY/open
Registered share	100 (parts)/ 40 (clips)	94 [89–100], green band	100/100	100	44 [1–100] side/100 UK	0.8	–	0.7/100 (video)	3.2	5.0
Registration stability	3 of 3	1 of 1	3 of 3	2 of 2	4 of 5 side/0–2 of 5 front	1 of 1	–	2 of 2 video/1 of 1 stills	1 of 1	1 of 1
Models	1 [1–2]	1	1 [1–2]	1	2 [1–5]	1	–	1	1	1
Track length	6.0	5.2	5.2/8.3	4.2	4.0/4.4	–	–	5.0 (video)	–	–
Triangulation angle	6.4°	25.5°	9.7°/3.6°	10.3°	7.7/5.0°	–	–	6.2°	–	–
Viewpoint coverage	0.22 sr	1.50 sr	0.37/0.63 sr	0.35 sr	0.26/0.70 sr	–	–	0.37 sr	–	–
Blurred frames/ clipped pixels	0.0/0.1%	0.0/0.1%	0.0/1.1%	0.0/0.3%	0.0 [0–7]/0.2 [0–4]%, UK 0.0/12.3%	0.0/0.3%	–	15.7/0.2% (stills)	50.0/1.9%	0.0/2.3%
Static content	0.0%	0.0%	0.0%	0.0%	0 [0–34]%/1.2% UK	–	–	0.0%	–	–
Lens artifacts	none	vignetting (unsure)	glare/ghost (unsure)	not annotated	none, 2 passes not annotated	none	–	none	glare	none
Documentation reliability	parts not contiguous/ no trajectory	altitude/ panel counts	none (no trajectory, file naming)	log is GPS, not RTK	10 sessions, 11 listed	none (half of photos near trunks)	2 files without CRS	two licenses/ video in repo only	image count, dates, no license	image sizes/ views rotate bunches
